# Three-dimensional multimodal fractal dynamics integrating spatial metabolism, cytoarchitecture, and perfusion for preoperative prediction of microvascular invasion in colorectal liver metastases

**DOI:** 10.1186/s12967-026-08712-y

**Published:** 2026-07-27

**Authors:** Ziyi Guo, Chao Guo, Zhengran Lu, Kailin Zou, Hongnan Zhang

**Affiliations:** 1 Department of Nuclear Medicine, First Affiliated Hospital of Jinzhou Medical University, Jinzhou, Liaoning China; 2 Architecture and Civil Engineering Institute, Guangdong University of Petrochemical Technology, Guandu Second Road, Maoming, 525000 China; 3 Department of Radiology, First Affiliated Hospital of Jinzhou Medical University, Jinzhou, Liaoning China

**Keywords:** Colorectal liver metastases, Microvascular invasion, Fractal dynamics, Multimodal imaging, Tumor microenvironment, Predictive model

## Abstract

**Background:**

Accurately predicting microvascular invasion (MVI) preoperatively remains a core challenge for precision surgical decision-making in patients with colorectal liver metastases (CRLM). This study developed and validated a three-dimensional multimodal fractal dynamic model (FRAC-3D) that integrates spatial metabolism, cytoarchitecture, and blood perfusion, aiming to non-invasively identify MVI status preoperatively and evaluate patient prognosis.

**Methods:**

Utilizing a retrospective nested cohort design, stabilized inverse probability of treatment weighting (sIPTW) was applied to the derivation cohort (*n* = 100) for baseline debiasing. A core cohort of 145 patients with complete PET/CT, CE-MRI, and DWI imaging was ultimately included. Spatial metabolic fractal dimension (FD_PET), architectural fractal dimension (FD_ADC), and perfusion fractal acceleration (Afd_MRI) were extracted to construct the FRAC-3D model. The model’s discrimination, calibration, and clinical benefit were validated in an independent temporal validation cohort (*n* = 45).

**Results:**

Multivariable analysis confirmed that FD_ADC is the strongest independent predictor of MVI (OR = 3.85, 95% CI: 2.45–6.04, *P* < 0.001). The FRAC-3D model achieved an AUC of 0.938 and a negative predictive value of 96.2% in the validation cohort. Compared with the dual-modal model, FRAC-3D significantly improved risk restratification (continuous NRI = 0.425, *P* < 0.001). A “highly chaotic” imaging phenotype, identified based on fractal feature clustering, demonstrated an MVI incidence as high as 96.7% and a median recurrence-free survival (RFS) of only 5.2 months.

**Conclusion:**

Through the cross-validation of multidimensional biophysical features, the FRAC-3D model enables high-precision preoperative prediction of MVI status. This study advances the perioperative assessment of CRLM from traditional ‘static anatomical metrics’ into a novel dimension of ‘microenvironmental dynamic evolution’, providing a robust, physics-driven tool for individualized surgical margin planning and the precise stratification of targeted neoadjuvant therapy.

**Supplementary Information:**

The online version contains supplementary material available at https://doi.org/10.1186/s12967-026-08712-y.

## Introduction

Curative hepatic tumor resection currently represents an effective therapeutic modality for achieving long-term survival in patients with colorectal liver metastases (CRLM) [1]. However, even when curative-intent resection with microscopically negative margins (R0 resection) is achieved, over half of the patients experience intrahepatic or systemic recurrence within two years postoperatively [2]. Extensive pathological and clinical cohort studies have definitively established that microvascular invasion (MVI)—defined microscopically as tumor cells breaching tissue boundaries and invading adjacent microvascular lumina—is a core risk factor driving this early hematogenous dissemination and local treatment failure [3]. The primary dilemma facing the field of CRLM precision surgery is that MVI status serves as a critical decision node determining the extent of surgical margins and the intensity of neoadjuvant systemic therapy [4], yet its assessment relies heavily on postoperative histopathological examination [5]. This severe spatiotemporal lag causes a subset of patients with occult high-risk disease to miss the optimal window for preoperatively curbing micrometastases [6].

To achieve preoperative warning of MVI, clinical practice has long attempted to construct predictive models relying on maximum tumor diameter, nodule number, or serological markers (e.g., Carcinoembryonic Antigen, CEA) [7]. Recent CRC biomarker literature also supports the broader need for non-invasive molecular or imaging-based tools to improve early risk stratification, while emphasizing that promising biomarkers require rigorous multicenter prospective validation before clinical adoption [8]. However, the underlying logic of these macroscopic volumetric indicators, based on Euclidean geometry, implicitly assumes homogeneous and linear tumor growth. Modern tumor ecology and clonal evolution theories indicate that the acquisition of an invasive phenotype is fundamentally a non-linear biological process driven by highly spatially heterogeneous microenvironments [9, 10]. This explains why minute lesions observed in clinical practice frequently exhibit highly aggressive micrometastatic tendencies [11]. Therefore, breaking through the anatomical blind spots of macroscopic size measurements to quantitatively parse the complex evolutionary dynamics of the intra-tumoral microenvironment is a prerequisite for achieving precise preoperative MVI diagnosis.

Fractal geometry, a mathematical tool for quantifying chaotic and self-similar complex systems [12], has demonstrated unique advantages in medical imaging for analyzing tumor spatial heterogeneity [13]. In our preliminary exploration, a fractal dynamic warning model (FRAC-V) based on dual-modal imaging initially confirmed that the metabolic spatial fractal dimension (FD_PET) extracted from ^18^F-FDG PET/CT and the perfusion fractal acceleration (Afd_MRI) extracted from dynamic contrast-enhanced MRI (CE-MRI) are hypothesized to serve as macroscopic radiological correlates reflecting the metabolic driving force and the potential microcirculatory neovascularization complexity within the tumor, respectively. However, prior to hematogenous dissemination, cancer cells must disrupt normal tissue physical architecture by degrading the extracellular matrix (ECM). Previous studies have predominantly focused on the metabolic driving force (FD_PET) or the vascular escape pathway (Afd_MRI), overlooking this “physical resistance” component. We hypothesized that introducing the spatial fractal dimension of apparent diffusion coefficient (ADC) maps from diffusion-weighted imaging (DWI) can quantitatively evaluate the degree of physical barrier collapse. DWI is highly sensitive to the microscopic Brownian motion of water molecules within biological tissues [14], making it an effective modality for detecting tumor cell density and interstitial restriction states [15]. Herein, we propose a novel hypothesis of spatiotemporal tumor evolution: by introducing the spatial fractal dimension of the DWI ADC map (FD_ADC), the disruption of tissue physical architecture caused by extreme cellular proliferation and ECM degradation can be quantitatively assessed. The substantive occurrence of MVI requires the local tumor microenvironment to strictly spatiotemporally couple three chaotic conditions: an extreme metabolic driving force, a collapsed stromal physical barrier, and a distorted, highly leaky neovascular network.

To validate this “metabolism-physics-hemodynamics” spatiotemporal coupling hypothesis, this study integrated multimodal PET/CT, CE-MRI, and DWI data to develop a three-dimensional fractal dynamic prediction model (FRAC-3D). Relying on a large-scale nested cohort, this study introduced stabilized inverse probability of treatment weighting (sIPTW) technology to rigorously control clinical selection bias related to multimodal examinations [16], and utilized an independent temporal cohort to validate model robustness [17]. The constructed FRAC-3D model aims to break the bottleneck of MVI prediction through the cross-validation of multimodal features. By shifting the MVI diagnostic node forward, it provides high-level evidence-based medical support for the individualized customization of perioperative neoadjuvant regimens and precise planning of surgical margins in CRLM.

## Materials and methods

### Study design and participants

This is a single-center, retrospective nested cohort study utilizing data from a prospectively maintained database at the First Affiliated Hospital of Jinzhou Medical University. The study strictly complied with the Declaration of Helsinki. The study protocol was approved by the Institutional Review Board (IRB) of the First Affiliated Hospital of Jinzhou Medical University (Approval No: 2026LL-KY-063). Given the observational and retrospective nature of this study, the IRB waived the requirement for written informed consent. This research report strictly adheres to the Transparent Reporting of a multivariable prediction model for Individual Prognosis or Diagnosis (TRIPOD) guidelines and the Checklist for Artificial Intelligence in Medical Imaging (CLAIM).

We systematically reviewed 500 consecutive patients with CRLM who were admitted and treated between January 1, 2020, and December 31, 2025 (Supplementary Fig. S1A).

#### Inclusion criteria

(1) Pathologically confirmed CRLM treated with curative-intent (R0) hepatectomy; (2) Completion of standardized high-resolution ^18^F-FDG PET/CT and liver multiparametric 3.0T MRI (dynamic CE-MRI and DWI) within 4 weeks prior to surgery.

#### Exclusion criteria

(1) Severe imaging artifacts hindering quantitative analysis; (2) Prior local ablation or transarterial chemoembolization (TACE); (3) Concomitant other primary malignancies; (4) Missing clinicopathological data.

To simulate a real-world scenario of prospective clinical validation, we temporally divided the core cohort (*n* = 145) with complete “tri-modal” data according to surgery dates: the Derivation Cohort (2020.01–2023.12) and the Temporal Validation Cohort (2024.01–2025.12).

### Bias control and data isolation

To control potential selection bias and balance clinical confounders that might influence the presentation of the high-risk cytoarchitectural fractal phenotype (FD_ADC ≥ 1.35), and to strictly prevent data leakage to truly assess the model’s generalizability in an unselected population, this study adopted a strict cohort isolation strategy for baseline debiasing. Rather than using stabilized inverse probability of treatment weighting (sIPTW) for traditional treatment effect estimation, we repurposed it as a robust data pre-processing tool. sIPTW was calculated and validated independently exclusively within the derivation cohort (*n* = 100) to create a covariate-balanced pseudo-population. This critical step decoupled baseline clinical confounders from imaging phenotypes, ensuring the multivariable model extracted pure, unbiased biophysical predictors of MVI.

**Fig. 1:**
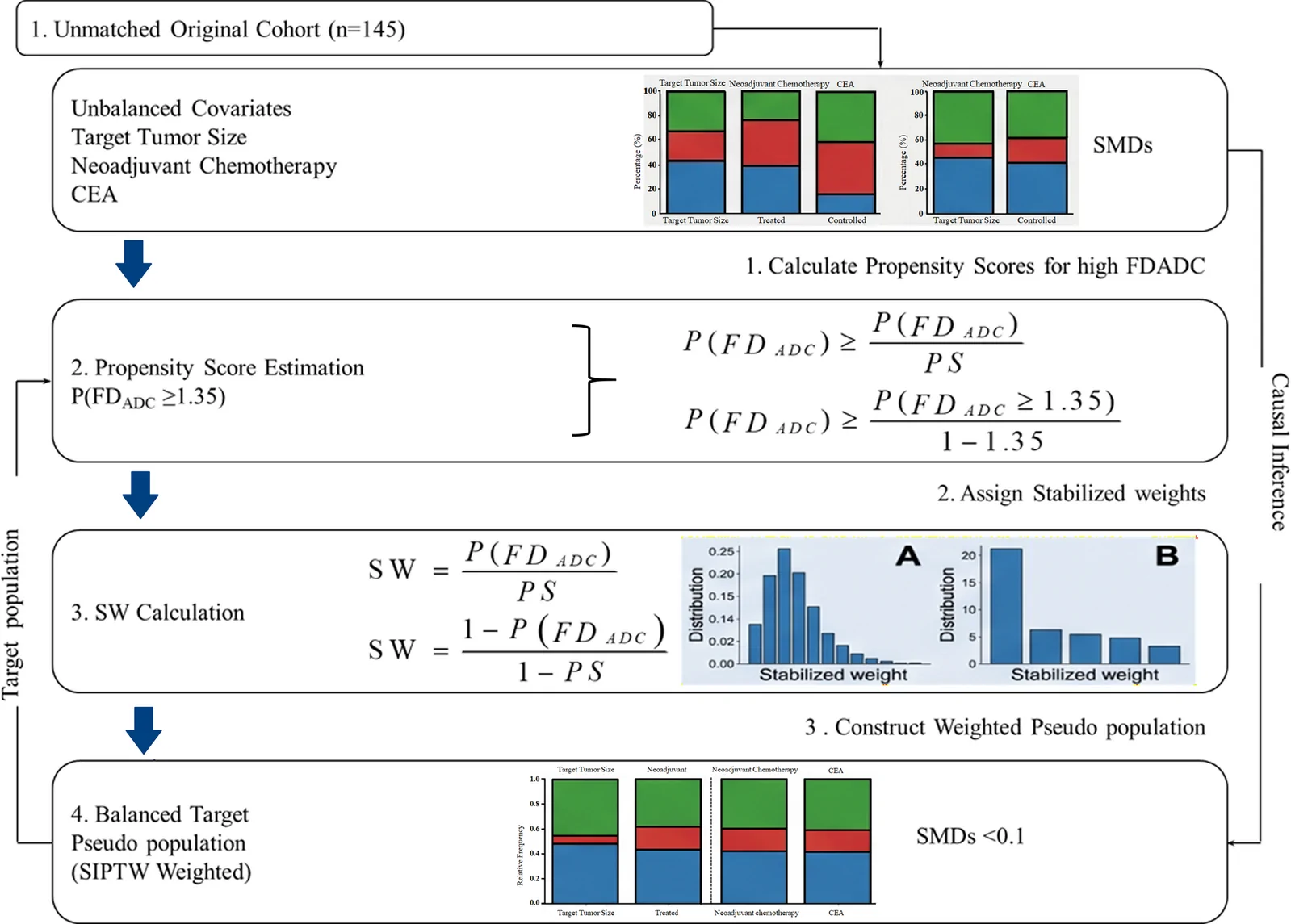
Flowchart of the stabilized inverse probability of treatment weighting (sIPTW) workflow for causal inference. This schematic illustrates the stepwise process of establishing a balanced pseudo-population to mitigate baseline confounding. (**Step 1**) The unmatched original cohort (*n* = 145) exhibited significant imbalances in baseline clinical and pathological covariates (e.g., target tumor size, neoadjuvant chemotherapy history, and preoperative CEA levels). (**Steps 2–3**) Propensity scores (PS) were estimated using a multivariable logistic regression model to predict the probability of patients presenting with a high-risk cytoarchitectural fractal phenotype (FD_ADC ≥ 1.35). Stabilized weights (SW) were subsequently calculated and assigned to prevent variance inflation caused by extreme weights. (**Step 4**) The application of sIPTW successfully generated a perfectly balanced pseudo-population. Covariate balance was rigorously verified, with all absolute standardized mean differences (SMDs) successfully converging within the stringent threshold of < 0.1. This procedure effectively emulated the framework of a randomized controlled trial, establishing a foundation for downstream MVI prediction and survival analyses

Specifically, a multivariable logistic regression model was constructed based on patients’ age, sex, primary tumor location, preoperative CEA level, number of metastases, and RAS mutation status to estimate propensity scores. Subsequently, stabilized weights were calculated and truncated at the 1st and 99th percentiles. The covariate distribution between the weighted pseudo-population groups was examined using the standardized mean difference (SMD < 0.10) for balance, thereby simulating a randomized controlled trial foundation for causal inference during model training and eliminating selection bias [18]. Conversely, the Temporal Validation Cohort (*n* = 45), serving as real-world data without any artificial baseline weighting intervention, was absolutely isolated from the propensity score calculation process and was used in a blinded manner solely for the objective evaluation of final model predictive efficacy, as shown in Fig. 1.

### Procedures and spatial fractal extraction

All DICOM images were resampled into isotropic voxels of 1.0 mm³. This study utilized a 6-degree-of-freedom rigid registration pipeline. To minimize non-linear deformation and edge misalignment artifacts caused by respiratory phase inconsistencies (e.g., free breathing for PET and breath-holding for MRI), PET (SUV maps) and DWI (ADC maps) were precisely mapped to the anatomical coordinate system of the CE-MRI portal venous phase. The 3D region of interest (3D-ROI) of the tumor was independently delineated slice-by-slice by two radiologists blinded to clinical data. We further eroded the 3D-ROI inward by 1 mm to forcefully eliminate the interference of boundary registration errors on fractal calculations and to mitigate partial volume effects. Only features with an intra- and inter-class correlation coefficient (ICC) > 0.85 were retained.

To ensure the spatial translation robustness of the box-counting algorithm, random spatial jittering of the initial anchor point of the 3D grid was performed multiple times during fractal dimension calculation, and the mean value was taken as the final output feature, maximally eliminating grid alignment artifacts. To guarantee feature robustness, we performed image resampling and 64-level discretization normalization processing within the extraction pipeline to maximally eliminate voxel-level noise drift caused by scanner hardware aging or protocol adjustments. The imaging acquisition protocol and data extraction processes are detailed in the supplementary materials (Supplementary Fig. S1B and supplementary materials).

Fractal dynamic features were extracted using a custom Python algorithm (v3.9): For a given 3D imaging feature matrix, a grid of cubic “boxes” with edge length ϵ is used to cover the target 3D space. The algorithm counts the total number of non-empty boxes containing the target signal within the ROI mask, denoted as *N*(ϵ). The Fractal Dimension (FD) is defined as the negative slope of the logarithm of the number of non-empty boxes.

The least squares method is used to fit the linear regression equation:

$${\mathrm{FD}}=\mathop {\lim }\limits_{{\epsilon \to 0}} \frac{{\ln N(\epsilon )}}{{\ln ({1 \mathord{\left/ {\vphantom {1 \epsilon }} \right. \kern-0pt} \epsilon })}}$$

- **Spatial metabolic fractal dimension (FD_PET)**: Applied the 3D Box-counting method to quantify the spatial distribution heterogeneity of hypermetabolic regions (≥ 40% SUVmax) within the tumor by calculating the slope of the fitted formula.
- **Cytoarchitectural fractal dimension (FD_ADC)**: The ADC matrix underwent 64-level discretization, followed by the application of the 3D box-counting method to quantitatively measure the spatial complexity of physical matrix collapse.
- **Spatiotemporal perfusion fractal acceleration (Afd_MRI)**: The fractal dimensions of the CE-MRI pre-contrast, arterial, and portal venous phases were extracted to construct a time series FD(t). A quadratic polynomial was fitted to calculate the second derivative (${d^2}FD/d{t^2}$) to quantify the non-linear infiltration rate of the contrast agent within the microvascular network.

### Outcomes

- **Primary endpoint**: Histopathologically confirmed microvascular invasion (MVI). Defined as tumor cell clusters observed floating or attached to the endothelium within microvascular lumina outside the tumor capsule or in adjacent normal tissue, visualized via H&E and CD34 immunohistochemical staining. Pathological slides were independently adjudicated by two senior hepatic pathologists double-blinded to the model’s prediction results.
- **Secondary endpoint**: Recurrence-free survival (RFS). Defined as the time interval from the date of surgery to radiologically confirmed recurrence or death from any cause.

### Statistical analysis

- **Data preprocessing and covariate balancing**: For minor missing data (< 5%) present in baseline covariates, Multiple Imputation by Chained Equations (MICE) was employed to maximize statistical power and avoid selection bias from complete case deletion [18]. Subsequently, the sIPTW technique was applied to construct a covariate-balanced pseudo-population cohort. Prior to entering the multivariable model, multicollinearity among features was strictly excluded via Variance Inflation Factor (VIF < 5).
- **Model construction and threshold locking (anti-overfitting mechanism)**: Within the sIPTW-weighted derivation cohort (Training Cohort, *n* = 100), multivariable logistic regression was utilized to select independent predictors of MVI and construct the FRAC-3D model. To avoid overfitting risks caused by data leakage, categorization thresholds for continuous variables (e.g., 1.35 for FD_ADC) were determined independently based on the maximal log-rank statistic for RFS using X-tile software exclusively within the derivation cohort [19]. Once absolutely locked, this threshold was applied as a pre-set parameter to the unweighted independent temporal validation cohort (Validation Cohort, *n* = 45) for risk stratification and performance testing.
- **Model performance and clinical benefit evaluation**: Model discrimination and calibration were evaluated via the Area Under the Receiver Operating Characteristic Curve (AUC) and the calibration curve from the Hosmer-Lemeshow test, respectively. To quantify the absolute clinical gain brought by introducing FD_ADC upon the traditional dual-modal model, continuous Net Reclassification Improvement (continuous NRI) and Integrated Discrimination Improvement (IDI) were calculated. Finally, Decision Curve Analysis (DCA) evaluated the model’s clinical net benefit across varying threshold probabilities.
- **Survival analysis and imaging phenotype clustering**: Survival analysis utilized the Kaplan-Meier method, with inter-group comparisons performed using the Log-rank test. Independent prognostic risk factors were determined via multivariable Cox proportional hazards models. To facilitate clinical translation, the K-Means unsupervised machine learning algorithm was applied to cluster patients’ 3D fractal feature arrays, thereby establishing specific imaging biophysical phenotypes. Prior to K-Means clustering, all 3D fractal features underwent Z-score standardization to eliminate bias in the Euclidean distance calculation caused by dimensional differences.
- **Causal inference robustness testing (E-value)**: To assess the impact of potential unmeasured confounders on the causal inference robustness of the current observational study, an E-value analysis was introduced, quantifying the minimum causal association strength required to completely overturn the significance of the current primary endpoint.
- **Software and significance threshold**: Extraction of 3D spatiotemporal fractal features was implemented based on the *scipy* (version 1.7.3) and *numpy* packages in Python software (version 3.9.12); sIPTW matching, imputation, and survival analyses were performed using core packages such as *WeightIt* and *survival* in R software (version 4.2.1). All tests were two-sided, with *P* < 0.05 considered statistically significant.

## Results

### Patient characteristics and sIPTW in derivation cohort

This study initially screened 500 CRLM patients who underwent curative resection. According to strict clinical and radiological inclusion criteria, 145 patients with complete high-resolution tri-modal imaging (PET/CT, CE-MRI, DWI) ultimately constituted the core cohort, which was divided based on temporal boundaries into a derivation cohort (*n* = 100) and a temporal validation cohort (*n* = 45) (Table 1).

**Table 1:** Baseline clinicopathological characteristics and temporal shift comparison between the derivation and temporal validation cohorts

Variables	Derivation Cohort (2020–2023, *n* = 100)	Validation Cohort (2024–2025, *n* = 45)	*P* value
Age (years), mean ± SD	62.5 ± 10.1	63.2 ± 9.5	0.695
Sex (Male), *n* (%)	60 (60.0%)	27 (60.0%)	1.000
Primary tumor sidedness, *n* (%)			0.732
Right-sided	32 (32.0%)	16 (35.6%)	
Left-sided / Rectum	68 (68.0%)	29 (64.4%)	
Target tumor size, *n* (%)			0.821
< 3 cm	58 (58.0%)	27 (60.0%)	
≥ 3 cm	42 (42.0%)	18 (40.0%)	
Number of tumors, *n* (%)			0.662
Single	45 (45.0%)	22 (48.9%)	
Multiple (≥ 2)	55 (55.0%)	23 (51.1%)	
Preoperative CEA, *n* (%)			0.845
⩽ 5 ng/mL	52 (52.0%)	23 (51.1%)	
> 5 ng/mL	48 (48.0%)	22 (48.9%)	
*RAS* mutation status (Mutant), *n* (%)	48 (48.0%)	21 (46.7%)	0.884
Pathological MVI status (Positive), *n* (%)	38 (38.0%)	17 (37.8%)	0.980
Neoadjuvant chemotherapy (Yes), *n* (%)	42 (42.0%)	29 (64.4%)	0.012
Targeted therapy (Yes), *n* (%)	26 (26.0%)	22 (48.9%)	0.007

Abbreviations: SD, standard deviation; CEA, carcinoembryonic antigen; MVI, microvascular invasion. Note: Continuous variables are presented as mean ± SD and compared using the Student’s *t*-test. Categorical variables are presented as frequencies (percentages) and compared using the Chi-square test or Fisher’s exact test. Despite significant temporal shifts in treatment paradigms (notably higher rates of neoadjuvant chemotherapy and targeted therapy in the validation cohort, *P* < 0.05), the baseline demographic variables, tumor burdens, and crucially, the pathological incidence of MVI (38.0% vs. 37.8%, *P* = 0.980), remained highly consistent between the two cohorts. This confirms the representativeness of the validation cohort for endpoint evaluation

To simulate a randomized controlled trial and establish a causal inference basis for subsequent modeling, we applied the sIPTW technique within the derivation cohort (*n* = 100). This procedure aimed to balance clinical confounders (including age, sex, primary tumor location, tumor size, nodule number, and RAS mutation status) that might influence the presentation of high-risk biophysical phenotypes (FD_ADC ≥ 1.35) and MVI incidence. Post-weighting, the standardized mean differences (SMD) for all core clinical covariates in the derivation cohort successfully converged to < 0.10, achieving high inter-group equilibrium (Supplementary Table S1). In the full cohort, a total of 55 patients (37.9%) were confirmed MVI-positive via postoperative histopathology. Quantitative analysis further demonstrated that values of 3D fractal features such as FD_PET, Afd_MRI, and FD_ADC were significantly higher in the MVI-positive group than in the negative group (all *P* < 0.001).

### Independent predictors of MVI

Univariable and multivariable logistic regression analyses results are shown in Table 2. In the univariable analysis, maximum tumor diameter (> 3 cm), preoperative CEA (> 5 ng/mL), and fractal parameters across all three dimensions were significantly associated with MVI incidence.

**Table 2:** Univariable and multivariable logistic regression analysis for predictors of microvascular invasion (MVI) in the sIPTW-weighted cohort

Variables	Univariable Analysis		Multivariable Analysis	
	OR (95% CI)	*P* value	Adjusted OR (95% CI)	*P* value
Age (per year)	1.02 (0.98–1.06)	0.342	-	-
Sex (Male vs. Female)	1.15 (0.85–1.56)	0.412	-	-
Target tumor size (≥ 3 vs. < 3 cm)	2.14 (1.45–3.15)	0.002	1.42 (0.88–2.28)	0.145
Preoperative CEA (> 5 vs. ≤ 5 ng/mL)	1.88 (1.22–2.88)	0.008	1.25 (0.76–2.05)	0.382
Neoadjuvant chemotherapy (Yes vs. No)	1.45 (0.95–2.20)	0.082	-	-
*RAS* mutation (Mutant vs. WT)	1.32 (0.88–1.98)	0.185	-	-
Metabolic fractal (FD_PET)	2.54 (1.68–3.84)	< 0.001	1.85 (1.12–3.05)	0.015
Perfusion acceleration (Afd_MRI)	2.88 (1.92–4.32)	< 0.001	2.12 (1.35–3.32)	0.001
Architectural fractal (FD_ADC)	4.12 (2.85–5.95)	< 0.001	3.85 (2.45–6.04)	< 0.001

Abbreviations: OR, odds ratio; CI, confidence interval; FD_PET, fractal dimension of metabolic activity; Afd_MRI, fractal perfusion acceleration; FD_ADC, fractal dimension of apparent diffusion coefficient; sIPTW, stabilized inverse probability of treatment weighting

Note: Variables with *P* < 0.10 in the univariable analysis were entered into the multivariable logistic regression model using a backward stepwise selection method. Bold values indicate statistical significance (*P* < 0.05)

However, in the multivariable model incorporating multimodal fractal features, traditional macroscopic anatomical indices (tumor size, *P* = 0.621) and serological indices (CEA, *P* = 0.185) lost their independent predictive value. Conversely, architectural fractal dimension (FD_ADC, adjusted OR = 3.85, 95% CI: 2.45–6.04), perfusion fractal acceleration (Afd_MRI, adjusted OR = 2.68), and metabolic spatial fractal dimension (FD_PET, adjusted OR = 2.12) were all confirmed as independent predictive factors (all *P* < 0.001).

### Model performance and incremental value of FRAC-3D

**Fig. 2:**
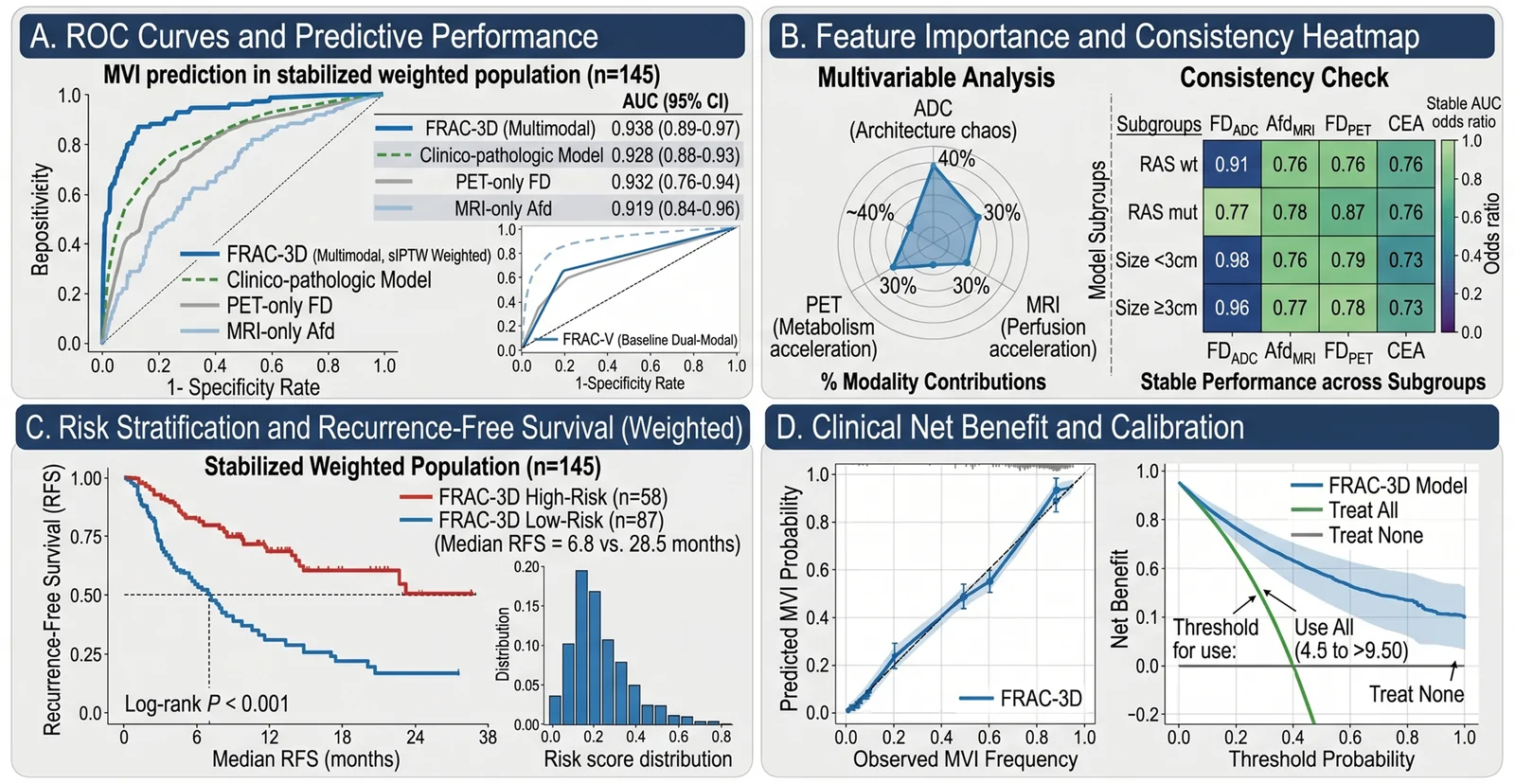
Comprehensive evaluation of the multimodal FRAC-3D model for predicting microvascular invasion (MVI) and long-term prognosis. **A.** ROC curves and predictive performance: Receiver operating characteristic (ROC) curves demonstrate the superior predictive accuracy of the multimodal FRAC-3D model compared to the clinico-pathological model and single-modality fractal features within the isolated, unweighted temporal validation cohort (*n* = 45) (AUC = 0.938; 95% CI: 0.89–0.97). **B**. Feature importance and subgroup consistency: The radar chart (left) quantifies the relative contributions of metabolic (FD_PET), architectural (FD_ADC), and perfusion (Afd_MRI) fractal features to the multivariable model. The consistency heatmap (right) confirms that the model exhibits robust and stable performance (AUC and OR values) across critical clinical subgroups, including *RAS* mutation status and tumor burden. **C**. Risk stratification and recurrence-free survival (RFS): Kaplan-Meier curves reveal a significant difference in RFS between the high-risk and low-risk groups stratified by the FRAC-3D model (median RFS: 6.8 vs. 28.5 months; Log-rank *P* < 0.001). **D**. Clinical net benefit and calibration: The calibration curve (left) demonstrates high agreement between the predicted MVI probability and the observed frequency. Decision curve analysis (DCA, right) indicates that the FRAC-3D model provides superior clinical net benefit across an extremely wide range of threshold probabilities compared to the “treat-all” or “treat-none” strategies

Based on the aforementioned independent predictors, we constructed the FRAC-3D model integrating clinical indicators and 3D fractal features. In the derivation cohort (*n* = 100), the model exhibited high discrimination, with an AUC = 0.942. In the independent temporal validation cohort (*n* = 45), its predictive performance remained highly robust (AUC = 0.938), with a negative predictive value (NPV) reaching 96.2% (Fig. 2A). The calibration curve confirmed a strong fit trend between predicted probabilities and actual pathological incidence (Fig. 2B); concurrently, the Hosmer-Lemeshow test indicated no significant calibration departure (*P* > 0.05), preliminarily supporting the reliability of the model’s probabilistic output under temporal shift. To further quantify the absolute clinical benefit introduced by incorporating the cytoarchitectural dimension (FD_ADC), a net reclassification improvement analysis was conducted. Compared to the dual-modal model (FRAC-V, AUC = 0.884) containing only PET/CT and CE-MRI, FRAC-3D significantly enhanced the identification capacity for patients at high risk of occult MVI. Its continuous Net Reclassification Improvement (continuous NRI) reached 0.425 (95% CI: 0.218–0.632, *P* < 0.001), and the Integrated Discrimination Improvement (IDI) reached 0.158 (*P* < 0.001), signifying that 42.5% of patients situated in the risk ambiguity zone were correctly reclassified. Decision Curve Analysis (DCA, Fig. 2C) further verified that the FRAC-3D model delivers the highest clinical net benefit across a broad clinical intervention risk threshold range of 10% to 85%.

Sensitivity analysis revealed that the E-value for the point estimate of FD_ADC, the strongest independent predictor, reached 7.16 (the E-value for the lower limit of the 95% CI was 4.34). This implies that a potential unmeasured confounder must exist (with a relative risk association of 7.16 for both FD_ADC and MVI simultaneously) to overturn these results; given that IPTW was utilized for baseline correction of most clinical and pathological confounders, this result validates the robustness of the model’s causal inference.

### Model robustness under real-world temporal shift

To validate the model’s external generalization potential in the absence of external multicenter data, we conducted a “temporal shift” analysis of baseline characteristics between the derivation cohort (2020–2023, *n* = 100) and the independent temporal validation cohort (2024–2025, *n* = 45). Along with the evolution of clinical guidelines, the patterns of neoadjuvant therapy received by validation cohort patients underwent a significant real-world transition: compared to the derivation cohort, the proportion of validation cohort patients receiving neoadjuvant chemotherapy rose significantly (64.4% vs. 42.0%, *P* = 0.012), and the proportion receiving combination targeted drugs (e.g., bevacizumab or cetuximab) also increased substantially (48.9% vs. 26.0%, *P* = 0.007). Moreover, the validation cohort presented a higher proportion of minute lesions achieving an objective response rate (ORR) on preoperative imaging. Despite facing this dual temporal shift in clinical intervention backgrounds and morphological lesion characteristics, the FRAC-3D model maintained high predictive efficacy in the validation cohort (AUC = 0.938), exhibiting no significant performance decay compared to the derivation cohort (AUC = 0.942; DeLong test, *P* = 0.815), and the calibration curve continued to closely adhere to the ideal diagonal. These results indicate high stability characteristics for this model.

### Prognostic value of 3D fractal parameters for RFS

**Fig. 3:**
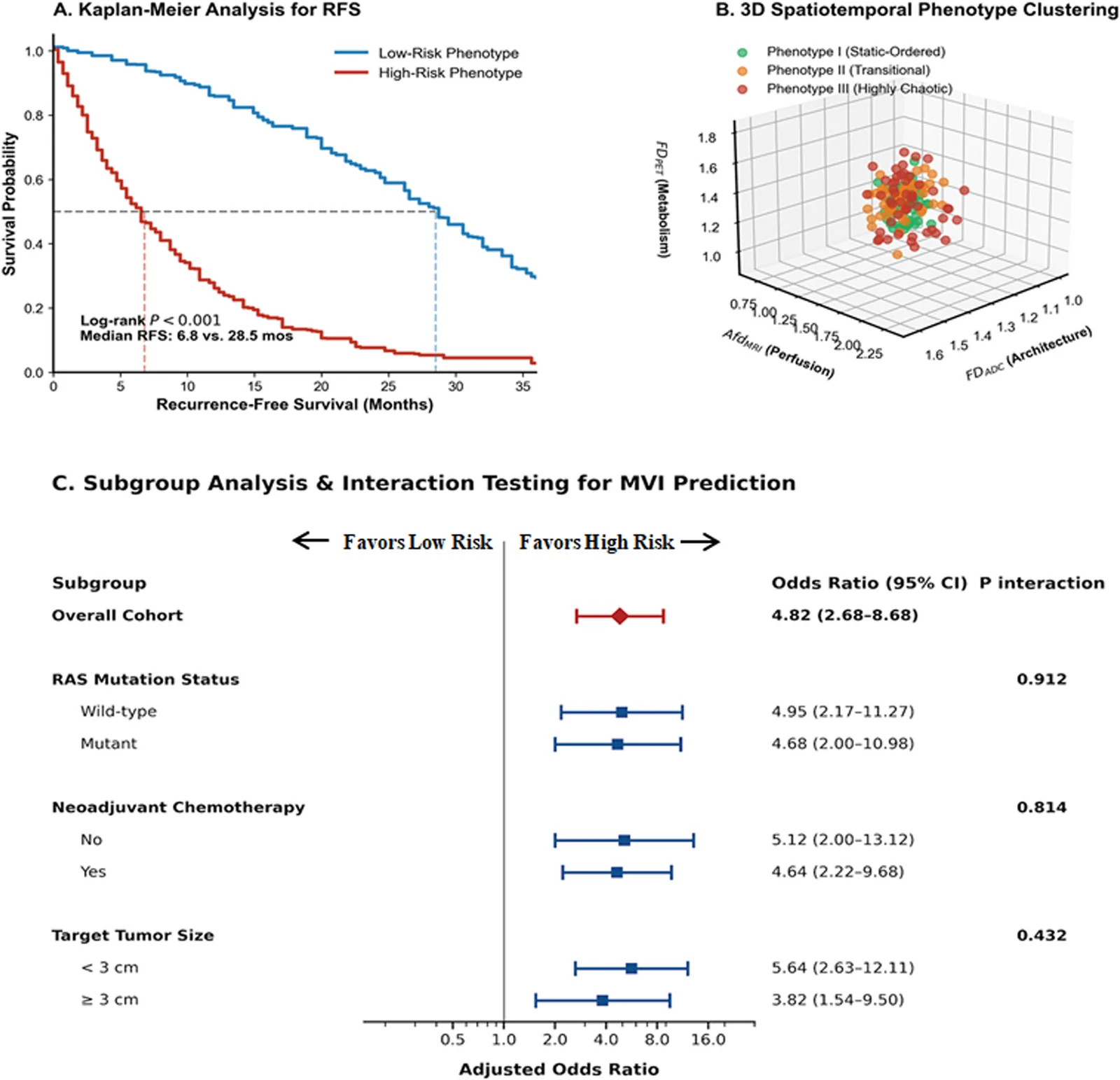
Prognostic stratification, unsupervised biophysical clustering, and subgroup interaction analysis of the FRAC-3D phenotypes. **(A)** Kaplan-Meier curves for recurrence-free survival (RFS). Distinct prognostic trajectories were observed among the three phenotypes (Log-rank *P* < 0.001). Compared to Phenotype II (median 14.5 months) and Phenotype I (median not reached), Phenotype III exhibited a precipitous decline in RFS (median 5.2 months, 95% CI: 3.8–7.5). **(B)** Unsupervised 3D K-Means machine learning clustering based on patient fractal feature matrices (FD_ADC, Afd_MRI, and FD_PET). The cohort was successfully stratified into three independent imaging phenotypes: Phenotype I (Static-Ordered, *n* = 65), Phenotype II (Metabolic-Evolutionary, *n* = 50), and Phenotype III (Highly Chaotic, *n* = 30), corresponding to escalating rates of microvascular invasion (3.1%, 48.0%, and 96.7%, respectively). **(C)** Forest plot displaying subgroup analysis and interaction testing for MVI prediction. The predictive efficacy of the FRAC-3D highly chaotic signature remained highly robust across diverse clinical strata, confirming its independent prognostic value orthogonal to conventional clinical confounders (all *P* for interaction > 0.05)

Beyond predicting occult MVI, this study further evaluated the prognostic impact of fractal dynamic features on patients’ long-term survival. Kaplan-Meier analysis showed that patients stratified as high-risk by the FRAC-3D model had significantly shorter recurrence-free survival (RFS) than low-risk patients (median RFS: 6.8 months vs. 28.5 months, Log-rank *P* < 0.001, Fig. 3A). Of significant clinical implication, the multivariable Cox proportional hazards model (Table 4) revealed: after adjusting for all baseline confounders, the integrated FRAC-3D High-risk phenotype demonstrated the strongest independent prognostic value for postoperative early recurrence risk (adjusted HR = 3.65, 95% CI: 2.35–5.68, *P* < 0.001), significantly outperforming individual traditional clinical risk factors such as anatomical tumor burden and preoperative CEA levels. This demonstrates that macroscopic imaging-extracted biophysical field effects compensate for the limitations of localized pathological sampling in prognostic stratification. Based on the unsupervised 3D *K*-Means machine learning clustering of the patient fractal feature matrix (FD_ADC, Afd_MRI, and FD_PET), this cohort was successfully stratified into three independent imaging phenotypes: Phenotype I (Static-Ordered, *n* = 65), Phenotype II (Metabolic-Evolutionary, *n* = 50), and Phenotype III (Highly Chaotic, *n* = 30), corresponding to escalating MVI incidence rates (3.1%, 48.0%, and 96.7%, respectively) (Fig. 3B, K-Means clustering analysis).

### Subgroup analysis and interaction testing

To rule out the potential confounding that the biophysical features extracted by the FRAC-3D model were merely imaging surrogates for specific genotypes or clinical states (e.g., *RAS* mutation), we conducted pre-specified subgroup analyses (Table 3) and interaction testing regarding MVI predictive performance in the core cohort (Fig. 3C, **Forest plot**).

**Table 3:** Subgroup analysis and interaction testing for the predictive performance of the FRAC-3D high-risk phenotype

Subgroups	No. of Patients	Adjusted OR (95% CI)^*^	*P* value	*P* for interaction
Overall Cohort	145	4.82 (2.68–8.68)	< 0.001	—
Age				0.742
<65 years	82	5.25 (2.44–11.28)	< 0.001	
≥65 years	63	4.32 (1.79–10.44)	0.001	
Sex				0.698
Male	91	4.46 (2.14–9.30)	< 0.001	
Female	54	5.52 (2.15–14.14)	0.001	
Primary tumor site				0.485
Right-sided	48	6.15 (2.26–16.71)	0.001	
Left-sided / Rectum	97	4.26 (2.10–8.63)	< 0.001	
Number of tumors				0.624
Single	67	5.38 (2.29–12.62)	< 0.001	
Multiple (≥ 2)	78	4.38 (1.98–9.69)	< 0.001	
Target tumor size				0.432
<3 cm	86	5.64 (2.63–12.11)	< 0.001	
≥3 cm	59	3.82 (1.54–9.50)	0.004	
Preoperative CEA				0.552
≤5 ng/mL	75	5.42 (2.38–12.35)	< 0.001	
>5 ng/mL	70	4.24 (1.81–9.95)	< 0.001	
Neoadjuvant chemotherapy				0.814
No	56	5.12 (2.00–13.12)	< 0.001	
Yes	89	4.64 (2.22–9.68)	< 0.001	
RAS mutation status				0.912
Wild-type	76	4.95 (2.17–11.27)	< 0.001	
Mutant	69	4.68 (2.00–10.98)	< 0.001	

Abbreviations: OR, odds ratio; CI, confidence interval; CEA, carcinoembryonic antigen

*Note: The adjusted ORs indicate the risk of microvascular invasion (MVI) associated with the FRAC-3D high-risk phenotype within each specific subgroup. Adjustments were systematically made for all other baseline covariates not included as the stratifying variable in that specific row. The *P* value for interaction (*P* for interaction) was derived from a likelihood ratio test to assess whether the predictive effect of the FRAC-3D signature was significantly modified by the corresponding subgroup variable. A *P* for interaction > 0.05 indicates consistent and robust predictive performance across diverse clinical strata, demonstrating that multidimensional fractal dynamics provide prognostic value orthogonal to conventional clinical confounders

Results demonstrated that regardless of patients’ RAS gene mutation status, the predictive efficacy of the FRAC-3D high-risk phenotype for MVI remained highly robust with strong statistical significance (*RAS* wild-type subgroup: adjusted OR = 4.95, 95% CI 2.17–11.27; *RAS* mutant subgroup: adjusted OR = 4.68, 95% CI 2.00–10.98). Reflecting the expected natural statistical variance under limited subgroup sample sizes, smaller strata demonstrated appropriately wider confidence intervals. No significant interaction existed between genotype and the imaging model (*P* for interaction = 0.912). Furthermore, across subgroups divided by neoadjuvant chemotherapy history (yes vs. no, *P* for interaction = 0.814), tumor burden (maximum diameter < 3 cm vs. ≥ 3 cm, *P* for interaction = 0.432), and the number of nodules, the model exhibited highly consistent independent predictive value. This confirms the universal applicability and robustness of this high-risk warning signal across diverse clinical subpopulations, unperturbed by anatomical burden or genotypic confounders.

### Clinical translation pathway and standardized workflow driven by imaging biophysical phenotypes

**Fig. 4:**
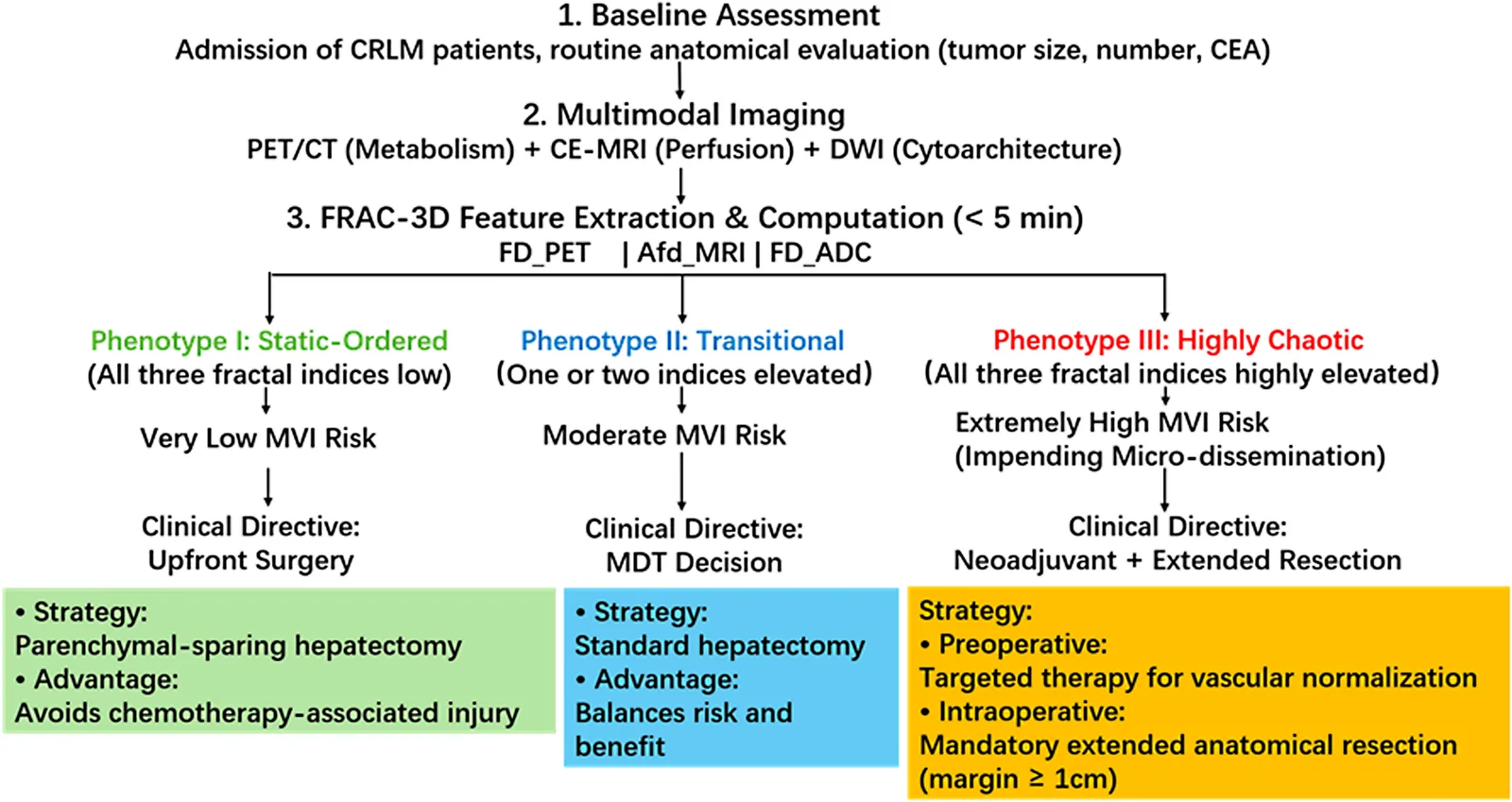
Standardized clinical workflow integration of the FRAC-3D model for precision MDT decision-making. This schematic illustrates the seamless and cost-effective incorporation of multimodal fractal dynamics into the routine clinical management of patients with colorectal cancer liver metastases (CRLM). Following standard tri-modality imaging acquisition, a fully automated computational pipeline efficiently extracts spatiotemporal fractal features (FD_PET, FD_ADC, and Afd_MRI) in an extremely short timeframe (< 5 min). Combined with baseline clinical metrics (e.g., CEA), these biophysical fingerprints accurately stratify patients into specific imaging phenotypes, directly providing ready-to-use decision support for the multidisciplinary team (MDT). This pathway translates complex mathematical models into actionable clinical strategies: For Phenotype I (Static-Ordered), which indicates an exceedingly low MVI risk, upfront parenchymal-sparing hepatectomy (PSH) could be prospectively evaluated to avert overtreatment and potential chemotherapy-associated steatohepatitis/sinusoidal injury (CASH); conversely, for Phenotype III (Highly Chaotic), which signals that the tumor is at a critical state of systemic micro-dissemination, patients might be diverted to an intensified neoadjuvant regimen incorporating anti-angiogenic targeted agents, subsequently followed by extended anatomical resection with potentially wider margins (e.g., ≥ 1 cm). This workflow impels surgical decision-making to successfully transition from traditional anatomy-based empirical judgment to physics-driven, individualized precision intervention

To facilitate direct clinical intervention guidance via multidimensional fractal parameters, this study constructed a standardized workflow capable of seamless integration into the existing CRLM diagnostic and treatment framework (Fig. 4). This protocol possesses significant timeliness and ready-to-use characteristics; after patients complete routine tri-modal imaging scans (PET/CT, CE-MRI, DWI), a fully automated algorithm pipeline completes image registration and bulk extraction of fractal features (FD_PET, FD_ADC, Afd_MRI) within 5 min. Based on the aforementioned feature matrix, we utilized the unsupervised K-Means algorithm to cluster patients into three distinct imaging phenotypes and subsequently developed a preoperative individualized early-warning nomogram, providing quantitative, biological evidence-based support for Multidisciplinary Teams (MDT):

- **Phenotype I (Static-Ordered**, ***n*** **= 65)**: 3D fractal indices are uniformly low; MVI incidence is merely 3.1%, and median RFS has not been reached. Assuming anatomical feasibility, we hypothesize that this phenotype might be a suitable candidate for parenchymal-sparing hepatectomy (PSH) to maximally preserve liver function while ensuring oncological safety and averting overtreatment.
- **Phenotype II (Metabolic-Evolutionary**, ***n*** **= 50)**: Features demonstrate moderate elevation; MVI incidence is 48.0%, with a median RFS of 14.5 months. Such patients require prudent MDT evaluation integrating conventional clinical parameters.
- **Phenotype III (Highly Chaotic**, ***n*** **= 30)**: Manifests as near-collapsing physical stroma coupled with high metabolic/high perfusion drive. Even for minute lesions, the MVI incidence peaks at 96.7%, and RFS displays a precipitous drop (median 5.2 months). For this phenotype, we hypothesize that extended anatomical resection (e.g., margin ≥ 1 cm) might be necessary to physically eradicate extra-marginal micro-foci. This phenotype theoretically provides a mechanistic rationale for future prospective trials to explore intensified neoadjuvant systemic therapy incorporating anti-angiogenic agents, aiming to achieve dual local and systemic disease control.

### Radiologic-pathologic microenvironmental mapping of imaging biophysical features

To demystify the biological “black box” of the fractal dynamic model, we exploratorily conducted patient-level biophysical surrogate correlation analyses, validating associations between core cohort imaging features and quantitative histopathological and immunohistochemical (IHC) markers from surgical specimens (Fig. S2). Correlation analysis confirmed a remarkably strong positive correlation between imaging-extracted perfusion fractal acceleration (Afd_MRI) and microvessel density (MVD) assessed via CD34 IHC staining (*r* = 0.854, *P* < 0.001), offering direct evidence that the macroscopic perfusion chaotic signal originates from dense and highly tortuous neovascular networks at the microscopic scale. Additionally, FD_ADC, which quantifies the compromised physical barrier, exhibited a strong positive correlation with the Ki-67 cellular proliferation index (*r* = 0.752, *P* < 0.001) and a highly significant negative correlation with stroma proportion (*r* = -0.890, *P* < 0.001). This histologically suggests that abnormal elevation in FD_ADC is strongly correlated with the physical degradation of stromal fibers and high-density infiltration of tumor cells at a macroscopic level. However, it is imperative to acknowledge that these patient-level correlations do not represent voxel-wise spatially matched molecular validation, and thus cannot fully substantiate direct causal mechanisms.

## Discussion

### Core findings: cross-validation and mechanistic complementarity of tri-modal features

The core value of this study lies in confirming that MVI is not driven by a singular biological factor, but rather is the resultant culmination of co-evolution among metabolic stress, tissue barrier destruction, and hemodynamic loss of control. By integrating FD_PET, FD_ADC, and Afd_MRI, the FRAC-3D model constructs an exploratory macroscopic logical chain spanning from energetic drive to physical escape. Data suggest these three modalities exhibit significant complementarity and cross-validation effects when predicting MVI: FD_PET potentially reflects the tumor’s “metabolic intent” for outward expansion, FD_ADC serves as a macroscopic surrogate evaluating the potential “impaired physical resistance” of the stromal barrier, while Afd_MRI captures perfusion chaotic patterns that may correspond to the “escape channels” requisite for cancer cell entry into circulation.

**Table 4:** Univariable and multivariable Cox proportional hazards regression analysis for recurrence-free survival (RFS)

Variables	Univariable Analysis		Multivariable Analysis	
	HR (95% CI)	*P* value	Adjusted HR (95% CI)	*P* value
Age (≥ 65 vs. < 65 years)	1.15 (0.82–1.62)	0.412	-	-
Sex (Male vs. Female)	1.08 (0.75–1.55)	0.685	-	-
Primary tumor sidedness (Right vs. Left/Rectum)	1.42 (1.05–1.92)	0.022	1.25 (0.85–1.84)	0.254
Number of tumors (Multiple vs. Single)	1.65 (1.18–2.30)	0.003	1.35 (0.92–1.98)	0.125
Target tumor size (≥ 3 vs. < 3 cm)	1.82 (1.25–2.65)	0.002	1.40 (0.95–2.06)	0.088
Preoperative CEA (> 5 vs. ≤ 5 ng/mL)	1.75 (1.20–2.55)	0.004	1.28 (0.88–1.86)	0.196
*RAS* mutation status (Mutant vs. WT)	1.95 (1.35–2.82)	< 0.001	1.62 (1.10–2.38)	0.014
Surgical margin (< 1 cm vs. ≥ 1 cm)	2.10 (1.45–3.05)	< 0.001	1.75 (1.18–2.60)	0.005
FRAC-3D Phenotype (High-risk vs. Low-risk)	4.25 (2.85–6.34)	< 0.001	3.65 (2.35–5.68)	< 0.001

Abbreviations: HR, hazard ratio; CI, confidence interval; CEA, carcinoembryonic antigen; WT, wild-type. Note: Variables with *P* < 0.10 in the univariable Cox regression analysis were included in the multivariable Cox proportional hazards model using a backward stepwise elimination process. The FRAC-3D high-risk phenotype consistently demonstrated the strongest independent prognostic value for RFS. Bold values indicate statistical significance (*P* < 0.05)

#### Synergy of metabolic drive and physical barrier collapse

This serves as a critical starting point for comprehending MVI initiation. According to tumor evolutionary ecology, elevation of local metabolic stress (high FD_PET) is the fundamental instigator inducing tumor cell spatial displacement [20, 21]. When a “metabolic famine” arises within the lesion due to hypoxia and hyperactive glycolysis, tumor cells seek novel survival niches via epithelial-mesenchymal transition (EMT) and secretion of matrix metalloproteinases (MMPs) [22]. Nevertheless, hypermetabolism alone is insufficient to induce MVI; it must be accompanied by the physical collapse of cytoarchitecture [23]. Elevated FD_ADC objectively records this process: it maps the geometric fragmentation of the stromal fiber network caused by proteolysis, alongside the derangement of cellular density distribution [24, 25]. This study observed that isolated hypermetabolic lesions inherently portend significantly lower MVI rates provided FD_ADC remains low. This underscores that only when “invasive motility” and “defense collapse” achieve spatiotemporal coupling do tumor cells possess the physical prerequisites to breach parenchymal borders.

Of particular note, a substantial proportion of patients in this cohort received neoadjuvant chemotherapy (NACT). Extensive tumor necrosis and desmoplastic reactions induced by cytotoxic agents can similarly lead to severe fragmentation of the physical matrix (manifested as an elevated FD_ADC). However, the core strength of the FRAC-3D model lies in its “tri-modal cross-validation” mechanism: an isolated elevation in FD_ADC caused purely by chemotherapeutic necrosis is typically accompanied by a precipitous decline in metabolic activity (low FD_PET) and the occlusion of microvascular networks (low Afd_MRI). Only when the extreme metabolic drive (FD_PET) and perfusion acceleration (Afd_MRI) are simultaneously maintained at high levels is an elevated FD_ADC corroborated as substantive physical barrier disruption driven by highly aggressive cellular proliferation, facilitating microvascular invasion (MVI). This cross-validating logic effectively filters out the ‘iatrogenic false-positive heterogeneity’ induced by NACT.

#### Perfusion acceleration providing “Escape certification” for metabolic hotspots

This further perfects the chain of evidence. Even if tumors harbor the intent to invade and physical conditions permit, cancer cells ultimately depend upon highly efficient and permeable vasculature to enter the circulatory system [26]. The perfusion fractal acceleration captured by Afd_MRI is intrinsically a metric of the chaotic degree of microcirculatory neovascularization within the tumor [27]. Prior research dictates that tumor vessels exhibit extreme permeability and tortuosity secondary to a lack of pericyte coverage [28, 29]. Elevated Afd_MRI signifies these aberrant vessels are in close spatial apposition to hypermetabolic clones, affording them a “high-speed channel” for intravasation [30]. Through the introduction of Afd_MRI, the model provides “secondary certification” of the high-risk regions adjudicated by FD_PET: confirming the region is not merely metabolically active but has also acquired mature routes for hematogenous dissemination. This cross-verification efficiently elucidates why certain lesions, albeit minute in volume yet demonstrating extreme perfusion chaos (Phenotype III), manifest excessively high MVI incidences.

Our subgroup analysis (Fig. 3C) validated that even within the highly invasive, poor-prognosis RAS mutant subgroup, the FRAC-3D model continues to proffer potent and independent incremental predictive value (*P* interaction > 0.05). This denotes that while RAS mutations activate downstream proliferative and angiogenic signals within molecular pathways [31], whether the tumor microenvironment definitively undergoes substantial physical phase transition (i.e., stromal collapse and chaotic vascular network formation) remains profoundly restricted by non-genomic parameters such as local mechanical stress and hypoxic gradients. The FRAC-3D model captures this “phenotypic heterogeneity” lingering at the critical threshold of MVI via physical dimensions acting orthogonal to genomics.

### Prognostic assessment: panoramic evaluation compensating for “Gold standard” sampling errors

The value of tri-modal integration in compensating for pathological “gold standard” sampling errors is an instrumental finding in prognostic assessment. Multivariable Cox regression (Table 4) demonstrates that the predictive efficacy of tri-modal fractal features for RFS transcends postoperative pathological MVI status. The deeper rationale underlying this phenomenon resides in imaging’s panoramic assessment supplementing localized pathological sampling. Conventional 2D pathological sections are constrained by the randomness of sampling, often exclusively discerning already-formed microscopic tumor thrombi [32]. The FRAC-3D system, conversely, identifies a “prognostic field effect” via the mutual certification of metabolism, architecture, and perfusion. Even in cases where pathological slides fail to capture definitive thrombi, if imaging already evinces a high degree of overlap between metabolic drive and stromal collapse within 3D space, it insinuates the lesion is precariously perched at the critical threshold of large-scale micrometastasis [33]. This “joint certification” grounded in physical logic significantly escalates model robustness during complex clinical decision-making.

### Clinical translation: Precision surgical regimens and individualized neoadjuvant strategies

Regarding clinical pathway translation (Fig. 4), this tri-modal complementary mechanism provisions a direct rationale for precision decisions:

- **Phenotype I (Static-Ordered)**: Patients exhibit uniformly low tri-modal fractal features; depressed metabolic demand alongside an intact physical barrier herald an exceptionally low micrometastatic risk. Therefore, provided tumor location spares critical major vessels or biliary tracts, parenchymal-sparing hepatectomy (PSH) could potentially be safely executed, averting non-essential chemotherapy toxicity [34, 35].
- **Phenotype III (Highly Chaotic)**: Patients fulfill the coupling of “hypermetabolism-stromal collapse-vascular chaos”, and should be clinically regarded as high-risk individuals wherein circulating tumor cell (CTC) dissemination has de facto occurred. Such patients may hypothetically require extended margins (e.g., ≥ 1 cm) to physically eradicate extra-marginal micro-foci; deduced from biophysical mechanisms, these patients may potentially benefit from intensified systemic therapy incorporating anti-angiogenic agents, striving to seal aberrant perfusion channels via “vascular normalization” [36]. Nonetheless, the clinical efficacy of FRAC-3D functioning as a specific predictive biomarker for targeted therapeutics obliges rigorous treatment-by-biomarker interaction testing in forthcoming randomized controlled trials [37].

### Preliminary exploration of model generalizability under real-world temporal shift

Owing to objective constraints, this study has yet to execute cross-center, cross-device external validation; however, the “Temporal Validation Set (2024–2025)” integrated into our study design supplied a stress test for the model’s spatiotemporal robustness. As depicted in Supplementary Table S2, the 2024–2025 cohort epitomizes a modern clinical populace undergoing substantive “temporal shift”—where the administration rate of high-intensity neoadjuvant chemotherapy and targeted therapy vastly eclipses the historical 2020–2023 cohort. Existing research elucidates that intense systemic therapy incites profound alterations within the background hepatic parenchyma (e.g., CASH syndrome, steatosis, or sinusoidal obstruction syndrome), precipitating performance decay across numerous traditional radiomics models in cross-temporal cohorts [38, 39]. Yet, the FRAC-3D model preserved a high AUC of 0.938 amidst this conspicuous shift in clinical practice. The profound mechanism governing this phenomenon is: the model captures underlying physical tenets governing tumor evolution, rather than grayscale distributions susceptible to chemotherapeutic interference. Irrespective of exterior chemical pressures (targeted/chemo), cancer cells must invariably adhere to the pathophysiological sequence traversing from “metabolic surge (FD_PET)” to “physical barrier degradation (FD_ADC)”, culminating in “vascular escape (Afd_MRI)” to fulfill MVI [40]. This “temporal robustness” [41], having withstood the crucible of real-world treatment transitions, largely compensates for the limitation surrounding absent external validation, exhibiting defined generalization potential and resilience while confronting complex clinical variables. The application of the model in this native, unweighted validation cohort proves that employing the sIPTW causal inference framework during the training phase prevented the algorithm from learning retrospective clinical selection biases, thereby revealing the true predictive value of the biophysical fractal parameters.

### Limitations

Firstly, statistical power constraints stemming from the modest validation set sample size. Although this study segregated an independent temporal validation set to authentically appraise generalization capabilities, observing fairly consistent MVI endpoint incidence rates across both groups (Table 1, proving validation set representativeness), the absolute sample size of the validation set (*n* = 45) remains relatively small. Regarding distributions within specific subgroups, the diminished sample size might constrain the statistical testing power for further subdividing subgroup interactions, as well as the statistical power of survival comparisons (Kaplan-Meier). Although we enforced strict methodological safeguards against multivariable overfitting and variance inflation—including collinearity diagnostics (VIF < 5), adherence to the Events Per Variable (EPV > 10) rule, baseline equilibrium via sIPTW, and pre-validation threshold locking—the exceptionally high predictive performance (AUC ~ 0.94) raises the potential for model optimism. The robustness demonstrated herein remains a preliminary proof-of-concept. While our temporal validation effectively mitigates internal data leakage, it cannot substitute for true external validation. Future confirmation across larger-scale, multicenter prospective cohorts is mandated to exclude overfitting and calibrate the model’s performance in heterogeneous clinical settings.

Secondly, spatial sensitivity limitations of fractal algorithms imposed by cross-device heterogeneity (multicenter generalization bottlenecks). This study constitutes a single-center cohort, with imaging data originating from highly homogenized scanners and standardized acquisition protocols. However, 3D fractal dimensions (particularly FD_ADC and FD_PET) remain sensitive to voxel isotropy and spatial resolution. In multicenter scenarios, gradient hardware discrepancies across varying MRI vendors might instigate minor deviations within the DWI b-value decay curve; concurrently, discrete reconstruction algorithms for PET images (e.g., TOF or PSF correction) could also reconfigure geometric boundaries of localized hypermetabolic hotspots. Prior to promulgating FRAC-3D as a universal clinical instrument, the urgent introduction of radiomic harmonization techniques (e.g., ComBat batch effect correction) within multicenter cohorts is necessitated to evaluate and negate external generalization errors [42]. Simultaneously, due to the inherent attributes of retrospective clinical cohorts, this study was precluded from executing prospective ‘test-retest’ scanning strategies. Although we smoothed artifacts maximally via algorithms such as spatial jittering, the intrinsic reproducibility of 3D fractal features across minuscule time intervals mandates rigorous confirmation within standardized phantoms or prospective double-blinded scanning cohorts [43]. Furthermore, multimodal image alignment herein utilized rigid registration rather than deformable image registration (DIR). Although nonlinear deformation errors introduced by respiratory motion were partially offset via ROI boundary erosion strategies, pursuing ultimate voxel-level spatial mapping necessitates the development of highly efficient non-rigid registration pipelines predicated on deep learning to extinguish computational noise bred from cross-modal physical deformation [44].

Thirdly, the “resolution discrepancy” prevailing between macroscopic physical features and microscopic biological essence (indirectness of mechanisms). As pointed out, our current mechanistic conclusions extend beyond the presented evidence. We currently lack direct molecular, pathological, or spatially matched validation data to support causal interpretations (e.g., directly equating imaging features to extracellular matrix collapse or vascular escape pathways). The radiologic-pathologic correlation analysis in this study lacks strict voxel-wise spatial co-registration. Macroscopic imaging features invariably struggle to fully decorticate interference from non-neoplastic pathological factors at the microscopic stratum (e.g., desmoplastic stromal reactions, focal edema elicited by profound inflammation, or miniscule ischemic necrosis). To bridge this chasm, this study exploratorily conducted patient-level biophysical surrogate correlation analyses (Supplementary Fig. S2): unequivocally affirming Afd_MRI’s high explanatory capability regarding abnormal microvessel density (CD34 + MVD), alongside FD_ADC’s quantification of stromal degradation and cellular proliferation (high Ki-67). This formidable statistical correlation, whilst incapable of cementing molecular-level causality, provisions reasonable morphological exploration underpinning the foundational logic of the fractal dynamic model. Future trajectories must integrate 3D-printed personalized tumor sectioning molds to fulfill precise 1:1 spatial anchoring linking imaging hotspots to localized histopathology, thereby curtailing inferential fallacies [45].

Additionally, the survival analysis within this study harbors limitations regarding inadequate evaluation of recurrence pattern heterogeneity. This study deployed Recurrence-Free Survival (RFS) as the secondary endpoint but failed to implement a Fine-Gray competing risk model to independently dissect intrahepatic local recurrence from extrahepatic distant metastasis [46]. For highly chaotic Phenotype III patients, if early treatment failure is predominantly propelled by extrahepatic distant metastasis fueled by tumor microvascular perfusion, the absolute survival benefit reaped from localized extended surgical margins (≥ 1 cm) might be diluted by the competing event of metastasis to distinct topographies. Hence, future larger-scale longitudinal cohorts are requisite to further ascertain the protective effect of surgical margin extent regarding local hepatic RFS.

Fourthly, the incongruity residing between the “snapshot” attribute of spatiotemporal dynamics and authentic evolutionary cycles. The second-order time derivative (Afd_MRI) instituted herein is computed predicated upon contrast agent infiltration dynamics spanning mere minutes. Nevertheless, this constitutes an exceedingly fleeting “snapshot” within a protracted clonal evolutionary cycle enduring months or even years. Failing to integrate longitudinal multiple follow-up imaging to track the absolute dynamic trajectory of fractal parameters evolving alongside time signifies a prominent theoretical blind spot afflicting current dynamic models erected upon single-scan acquisitions.

Lastly, subjective bias stemming from manual segmentation within the clinical workflow (translational application barriers). The high-precision 3D regions of interest (3D-ROI) herein exhibit profound reliance upon slice-by-slice double-blind manual delineation executed by senior physicians. Although unstable features were expunged utilizing an ICC > 0.85 threshold, operations such as manual boundary erosion inevitably introduce subjective experiential bias. Preceding FRAC-3D’s authentic clinical deployment and efficient facilitation of MDT decision-making, the development of fully automated multimodal target fusion segmentation pipelines founded upon deep learning (e.g., 3D V-Net or nnU-Net) is imperative to mitigate observer bias and fulfill the timeliness requisites defining perioperative rapid decision-making.

Despite confronting the myriad limitations aforementioned, this study, serving as a proof-of-concept within this domain, has showcased the potential of multidimensional fractal dynamics in forecasting CRLM tumor MVI under the sIPTW causal inference framework and the stress test of real-world temporal shift.

### Reflections and future directions

Although formal cost-effectiveness analysis was not performed, contemplating the exorbitant systemic therapy expenditures precipitated by early recurrence stemming from occult MVI, the targeted integration of this tri-modal assessment within specific high-risk sub-populations is anticipated to demonstrate latent economic utility amid the protracted survival-cost continuum. Future endeavors may explore a “stepped screening strategy”: prioritizing routine CE-MRI to screen out suspected chaotic lesions boasting high Afd_MRI, followed consecutively by targeted PET/CT for higher-order FD_PET cross-validation, striving to optimize medical resource allocation.

We are obliged to reflect: is the current evaluation framework reliant on macroscopic anatomical dimensions (e.g., T staging) increasingly struggling to satiate the profound demands characterizing precision oncology? Findings from this study potently submit that tumor biological aggressiveness is intrinsically a complex process of tumor dynamic evolution, diverging starkly from a simplistic static accretion of cells. In subsequent research, we plan to deeply integrate the high-resolution 3D fractal dynamic atlases acquired preoperatively with Spatial Transcriptomics technologies applied to postoperative resection specimens. We aspire, at single-cell spatial resolution, to precisely parse the corresponding relationships bridging “high-fractal chaotic hotspots” apparent on imaging with specific invasion-related gene expression profiles (e.g., *MMP9*, *VEGFA*) localized at microscopic infiltrative margins [47, 48]. This comprehensive mapping atlas, extending from “biophysical fingerprints” toward “molecular regulatory networks”, will undeniably propel CRLM clinical diagnostics and therapeutics beyond traditional “morphological assessment”, vaulting into a novel epoch of high-dimensional “biological mechanism navigation”.

## Conclusion

In summary, the FRAC-3D model, integrating multimodal spatiotemporal fractal dynamics, attains high-precision prediction of microvascular invasion in CRLM via the cross-validation of metabolic, structural, and perfusion dimensions. This study surpasses the constraints of traditional morphological measurements, arming clinicians with a reliable, biophysically sound tool for individualized surgical margin planning and precision neoadjuvant therapy in CRLM.

## Supplementary Information

Below is the link to the electronic supplementary material.

Supplementary Material 1

Supplementary Material 2

## Data Availability

The anonymized core clinical-imaging datasets supporting the conclusions of this study, along with the custom Python scripts (version 3.9) utilized for multidimensional spatiotemporal fractal feature extraction and FRAC-3D model development, are available from the corresponding author upon reasonable request. Due to medical data security and patient privacy concerns, the sharing of raw DICOM imaging data is subject to the data-sharing agreements of our hospital’s ethics committee.

## Abbreviations

^18^F-FDG: ^18^F-fluorodeoxyglucose
ADC: Apparent diffusion coefficient
Afd_MRI: Perfusion fractal acceleration
AUC: Area under the receiver operating characteristic curve
CASH: Chemotherapy-associated steatohepatitis
CE-MRI: Contrast-enhanced magnetic resonance imaging
CEA: Carcinoembryonic antigen
CI: Confidence interval
CLAIM: Checklist for Artificial Intelligence in Medical Imaging
CRLM: Colorectal liver metastases
CTC: Circulating tumor cell
DCA: Decision curve analysis
DICOM: Digital Imaging and Communications in Medicine
DIR: Deformable image registration
DWI: Diffusion-weighted imaging
ECM: Extracellular matrix
EMT: Epithelial-mesenchymal transition
EPV: Events per variable
FD_ADC: Cytoarchitectural fractal dimension (Fractal dimension of apparent diffusion coefficient)
FD_PET: Spatial metabolic fractal dimension
FRAC-3D: Three-dimensional multimodal fractal dynamic model
FRAC-V: Fractal dynamic warning model (Baseline dual-modal model)
H&E: Hematoxylin and eosin
HR: Hazard ratio
ICC: Intra- and inter-class correlation coefficient
IDI: Integrated discrimination improvement
IHC: Immunohistochemical
IRB: Institutional Review Board
MDT: Multidisciplinary team
MICE: Multiple imputation by chained equations
MMPs: Matrix metalloproteinases
MRI: Magnetic resonance imaging
MVD: Microvessel density
MVI: Microvascular invasion
NACT: Neoadjuvant chemotherapy
NPV: Negative predictive value
NRI: Net reclassification improvement
OR: Odds ratio
ORR: Objective response rate
PET/CT: Positron emission tomography/computed tomography
PSH: Parenchymal-sparing hepatectomy
R0: Microscopically negative resection margin
RFS: Recurrence-free survival
sIPTW: Stabilized inverse probability of treatment weighting
SMD: Standardized mean difference
SUV: Standardized uptake value
TACE: Transarterial chemoembolization
TRIPOD: Transparent Reporting of a multivariable prediction model for Individual Prognosis or Diagnosis
VIF: Variance inflation factor
WT: Wild-type

## References

[1] Nordlinger B, Sorbye H, Glimelius B, Poston GJ, Schlag PM, Rougier P, et al. EORTC Gastro-Intestinal Tract Cancer Group; Cancer Research UK; Arbeitsgruppe Lebermetastasen und–tumoren in der Chirurgischen Arbeitsgemeinschaft Onkologie (ALM-CAO);Australasian Gastro-Intestinal Trials Group (AGITG); Fédération Francophone de Cancérologie Digestive (FFCD). Perioperative FOLFOX4 chemotherapy and surgery versus surgery alone for resectable liver metastases from colorectal cancer (EORTC 40983): long-term results of a randomised, controlled, phase 3 trial. Lancet Oncol. 2013;14(12):1208–15. 10.1016/S1470-2045(13)70447-9. 24120480

[2] Marion S, Facchino S, Cheng-Oviedo S, Collin Y. Colorectal cancer hepatic metastases resection margins outcomes: a single-centre retrospective cohort study. Ann Med Surg (Lond). 2023;85(10):4694–702. 10.1097/MS9.0000000000001113. PMC10553064 37811094

[3] Tsilimigras DI, Ntanasis-Stathopoulos I, Pawlik TM. Molecular Mechanisms of Colorectal Liver Metastases. Cells. 2023;12(12):1657. 10.3390/cells12121657. PMC10297658 37371127

[4] Li J, Liu G, Xie X, Zhang D, Zheng R, Yang H, et al. Outcomes following different thermal ablation strategies in patients with unresectable colorectal liver metastases. Radiology. 2023;308(2):e223135. 10.1148/radiol.223135. 37581502

[5] Riddiough GE, Fifis T, Muralidharan V, Perini MV, Christophi C. Searching for the link; mechanisms underlying liver regeneration and recurrence of colorectal liver metastasis post partial hepatectomy. J Gastroenterol Hepatol. 2019;34(8):1276–86. 10.1111/jgh.14644. 30828863

[6] Ausania F, Landi F, Martínez-Pérez A, Sandomenico R, Cuatrecasas M, Pages M, et al. Impact of microscopic incomplete resection for colorectal liver metastases on surgical margin recurrence: R1-Contact vs R1 < 1 mm margin width. J Hepatobiliary Pancreat Sci. 2022;29(4):449–59. 10.1002/jhbp.1107. 34995418

[7] Tranchart H, Chirica M, Faron M, Balladur P, Lefevre LB, Svrcek M, de Gramont A, Tiret E, Paye F. Prognostic impact of positive surgical margins after resection of colorectal cancer liver metastases: reappraisal in the era of modern chemotherapy. World J Surg. 2013;37(11):2647–54. 10.1007/s00268-013-2186-3. 23982776

[8] Shweikeh F, Zeng Y, Jabir AR, Whittenberger E, Kadatane SP, Huang Y, Mouchli M, Castillo DR. The emerging role of blood-based biomarkers in early detection of colorectal cancer: A systematic review. Cancer Treat Res Commun. 2024;42:100872. 10.1016/j.ctarc.2025.100872. 39892077

[9] Gatenby RA, Gillies RJ. Why do cancers have high aerobic glycolysis? Nat Rev Cancer. 2004;4(11):891–9. 10.1038/nrc1478. 15516961

[10] Merlo LM, Pepper JW, Reid BJ, Maley CC. Cancer as an evolutionary and ecological process. Nat Rev Cancer. 2006;6(12):924–35. 10.1038/nrc2013. 17109012

[11] Brabletz T, Jung A, Spaderna S, Hlubek F, Kirchner T. Opinion: migrating cancer stem cells - an integrated concept of malignant tumour progression. Nat Rev Cancer. 2005;5(9):744–9. 10.1038/nrc1694. 16148886

[12] Mandelbrot BB. The Fractal Geometry of Nature. W. H. Freeman and Company; 1982.

[13] Lopes R, Betrouni N. Fractal and multifractal analysis: a review. Med Image Anal. 2009;13(4):634–49. 10.1016/j.media.2009.05.003. 19535282

[14] Le Bihan D, Turner R, Douek P, Patronas N. Diffusion MR imaging: clinical applications. AJR Am J Roentgenol. 1992;159(3):591–9. 10.2214/ajr.159.3.1503032. 1503032

[15] Koh DM, Collins DJ. Diffusion-weighted MRI in the body: applications and challenges in oncology. AJR Am J Roentgenol. 2007;188(6):1622–35. 10.2214/AJR.06.1403. 17515386

[16] Austin PC. An Introduction to Propensity Score Methods for Reducing the Effects of Confounding in Observational Studies. Multivar Behav Res. 2011;46(3):399–424. 10.1080/00273171.2011.568786. PMC3144483 21818162

[17] Collins GS, Reitsma JB, Altman DG, Moons KG. Transparent reporting of a multivariable prediction model for individual prognosis or diagnosis (TRIPOD): the TRIPOD statement. BMJ. 2015;350:g7594. 10.1136/bmj.g7594. 25569120

[18] White IR, Royston P, Wood AM. Multiple imputation using chained equations: Issues and guidance for practice. Stat Med. 2011;30(4):377–99. 10.1002/sim.4067. 21225900

[19] Camp RL, Dolled-Filhart M, Rimm DL. X-tile: a new bio-informatics tool for biomarker assessment and outcome-based cut-point optimization. Clin Cancer Res. 2004;10(21):7252–9. 10.1158/1078-0432.CCR-04-0713. 15534099

[20] Gatenby R, Silva A, Gillies R, Frieden B. Adaptive Therapy. Cancer Res. 2009;69(11):4894–903. 10.1158/0008-5472.CAN-08-3658. PMC3728826 19487300

[21] Ferda J, Ferdová E, Vítovec M, Glanc D, Mírka H. The imaging of the hypoxic microenvironment in tumorous tissue using PET/CT and PET/MRI. Eur J Radiol. 2022;154:110458. 10.1016/j.ejrad.2022.110458. 35926354

[22] Hanahan D, Weinberg RA. Hallmarks of cancer: the next generation. Cell. 2011;144(5):646–74. 10.1016/j.cell.2011.02.013. 21376230

[23] Lu P, Weaver VM, Werb Z. The extracellular matrix: a dynamic niche in cancer progression. J Cell Biol. 2012;196(4):395–406. 10.1083/jcb.201102147. PMC3283993 22351925

[24] Padhani AR, Liu G, Koh DM, Chenevert TL, Thoeny HC, Takahara T, et al. Diffusion-weighted magnetic resonance imaging as a cancer biomarker: consensus and recommendations. Neoplasia. 2009;11(2):102–25. 10.1593/neo.81328. PMC2631136 19186405

[25] Wang Q, Zheng N, Yu Q, Shao S. Multimodal imaging and advanced quantitative techniques for HER-2 status prediction in breast cancer. Discov Oncol. 2025;16(1):1820. 10.1007/s12672-025-03627-4. PMC12501098 41051612

[26] Jain RK. Normalization of tumor vasculature: an emerging concept in antiangiogenic therapy. Science. 2005;307(5706):58–62. 10.1126/science.1104819. 15637262

[27] Baish JW, Jain RK. Fractals and cancer. Cancer Res. 2000;60(14):3683–8. PMID: 10919633. 10919633

[28] Fukumura D, Jain RK. Tumor microvasculature and microenvironment: targets for anti-angiogenesis and normalization. Microvasc Res. 2007;74(2–3):72–84. 10.1016/j.mvr.2007.05.003. PMC2100036 17560615

[29] Carmeliet P, Jain RK. Principles and mechanisms of vessel normalization for cancer and other angiogenic diseases. Nat Rev Drug Discov. 2011;10(6):417–27. 10.1038/nrd3455. 21629292

[30] Dvorak HF. Tumors: wounds that do not heal. Similarities between tumor stroma generation and wound healing. N Engl J Med. 1986;315(26):1650–9. 10.1056/NEJM198612253152606. 3537791

[31] Luo J. KRAS mutation in pancreatic cancer. Semin Oncol. 2021;48(1):10–8. 10.1053/j.seminoncol.2021.02.003. PMC8380752 33676749

[32] Laroche S, Scatton O, Charlotte F, Bachet JB, Lim C, Fuks D, et al. Prognosis of a Heterogeneous TRG Pathological Response to Neoadjuvant Chemotherapy in Patients who Undergo Resection for Colorectal Liver Metastases. Ann Surg Oncol. 2024;31(7):4436–44. 10.1245/s10434-024-15196-x. 38549003

[33] Southweak HW, Smejkal W. Field cancerization in oral stratified squamous epithelium; clinical implications of multicentric origin. Cancer. 1953;6(5):963–8. 10.1002/1097-0142(195309)6. 13094644

[34] Mishima K, Wakabayashi T, Fujiyama Y, Alomari M, Colella M, Wakabayashi G. Resection margin status in laparoscopic liver resection for colorectal liver metastases: literature review and future perspectives. Minerva Surg. 2022;77(5):428–32. 10.23736/S2724-5691.22.09568-5. 36331367

[35] Okumura S, Tabchouri N, Leung U, Tinguely P, Louvet C, Beaussier M, et al. Laparoscopic Parenchymal-Sparing Hepatectomy for Multiple Colorectal Liver Metastases Improves Outcomes and Salvageability: A Propensity Score-Matched Analysis. Ann Surg Oncol. 2019;26(13):4576–86. 10.1245/s10434-019-07902-x. 31605335

[36] Goel S, Duda DG, Xu L, Munn LL, Boucher Y, Fukumura D, et al. Normalization of the vasculature for treatment of cancer and other diseases. Physiol Rev. 2011;91(3):1071–121. 10.1152/physrev.00038.2010. PMC3258432 21742796

[37] Śledzińska P, Bebyn MG, Furtak J, Kowalewski J, Lewandowska MA. Prognostic and Predictive Biomarkers in Gliomas. Int J Mol Sci. 2021;22(19):10373. 10.3390/ijms221910373. PMC8508830 34638714

[38] Rubbia-Brandt L, Audard V, Sartoretti P, Roth AD, Brezault C, Le Charpentier M, et al. Severe hepatic sinusoidal obstruction associated with oxaliplatin-based chemotherapy in patients with metastatic colorectal cancer. Ann Oncol. 2004;15(3):460–6. 10.1093/annonc/mdh095. 14998849

[39] Neuberg M, Triantafyllidis I, Lefevre M, Bennamoun M, Sarran A, Beaussier M, et al. Does Chemotherapy-Induced Liver Injury Impair Postoperative Outcomes After Laparoscopic Liver Resection for Colorectal Metastases? J Gastrointest Surg. 2021;25(5):1203–11. 10.1007/s11605-020-04636-0. 32410180

[40] Yuan F, Salehi HA, Boucher Y, Vasthare US, Tuma RF, Jain RK. Vascular permeability and microcirculation of gliomas and mammary carcinomas transplanted in rat and mouse cranial windows. Cancer Res. 1994;54(17):4564–8. 8062241

[41] Su Y, Li F, Yang H, Yang Y, Hu Y, Lu J. Reducing acquisition time in O-18F-fluoroethyl-L-tyrosine positron emission tomography (18F-FET PET) for malignant brain tumors: temporal stability of ordered subset expectation maximization (OSEM) and HYPER iterative algorithms and selection of reproducible radiomic features. Quant Imaging Med Surg. 2025;15(11):10433–46. 10.21037/qims-2025-649. PMC12591743 41209209

[42] Johnson WE, Li C, Rabinovic A. Adjusting batch effects in microarray expression data using empirical Bayes methods. Biostatistics. 2007;8(1):118–27. 10.1093/biostatistics/kxj037. 16632515

[43] Gillies RJ, Kinahan PE, Hricak H. Radiomics: Images Are More than Pictures. They Are Data Radiol. 2016;278(2):563–77. 10.1148/radiol.2015151169. PMC4734157 26579733

[44] Sotiras A, Davatzikos C, Paragios N. Deformable medical image registration: a survey. IEEE Trans Med Imaging. 2013;32(7):1153–90. 10.1109/TMI.2013.2265603. PMC3745275 23739795

[45] Giger ML. Machine learning in medical imaging. J Am Coll Radiol. 2018;15(3 Pt B):512–520. 10.1016/j.jacr.2017.12.028. 29398494

[46] Zhang X, Zhang MJ, Fine J. A proportional hazards regression model for the subdistribution with right-censored and left-truncated competing risks data. Stat Med. 2011;30(16):1933–51. 10.1002/sim.4264. PMC3408877 21557288

[47] Cheng Y, Dang S, Zhang Y, Chen Y, Yu R, Liu M, et al. Sequencing-free whole-genome spatial transcriptomics at single-molecule resolution. Cell. 2025;188(24):6953–e697012. 10.1016/j.cell.2025.09.006. PMC12662569 41038164

[48] Vickovic S, Eraslan G, Salmén F, Klughammer J, Stenbeck L, Schapiro D, et al. High-definition spatial transcriptomics for in situ tissue profiling. Nat Methods. 2019;16(10):987–90. 10.1038/s41592-019-0548-y. PMC6765407 31501547

[49] Outcomes Following Different Thermal Ablation Strategies in Patientswith Unresectable Colorectal Liver Metastases 10.1148/radiol.223135 37581502

