# Supplementary material for: Three-dimensional multimodal fractal dynamics integrating spatial metabolism, cytoarchitecture, and perfusion for preoperative prediction of microvascular invasion in colorectal liver metastases
Source: J Transl Med. 2026 Jul 27;24:1335. doi: 10.1186/s12967-026-08712-y (PMC13692898; doi:10.1186/s12967-026-08712-y)
Supplement: Supplementary file 1 [file 12967_2026_8712_MOESM1_ESM.pdf]

Supplementary Appendix:

Standard Operating Procedure (SOP) for 3D Fractal Dynamics Standardized Data Acquisition and Feature Extraction

This Standard Operating Procedure (SOP) applies to the acquisition, preprocessing, and quantitative calculation of preoperative multimodal imaging data for patients with colorectal liver metastases (CRLM). It aims to maximally eliminate technical biases caused by cross-device variations or subjective operator factors.

Part 1: Multimodal Imaging Underlying Acquisition Protocols and Hardware Parameters

1. 18F-FDG PET/CT Metabolic Spatial Acquisition

Acquisition Model: Integrated high-resolution PET/CT scanner (System default configuration: United Imaging PET/CT (uMI 510)).

Patient Preparation and Administration: Patients must fast for at least 6 hours, and capillary blood glucose must be ⩽ 7.0 mmol/L. The 18F-FDG radiotracer is injected intravenously via the median cubital vein at a dose of 3.7 - 5.5 MBq/kg based on the patient's body weight.

Acquisition Timing (Critical): The resting uptake time is strictly controlled to 60±5 minutes post-injection for whole-body and liver-targeted scanning.

Scanning and Reconstruction Parameters:

- **CT Attenuation Correction:** Utilizes a tube voltage of 120 kV, automatic tube current modulation (CareDose 4D, range 80-120 mAs), a pitch of 0.8, and a collimation of 0.6 mm. The reconstructed slice thickness is 3.0 mm.
- **PET Acquisition:** Adopts a three-dimensional (3D) List-mode acquisition, with a continuous scan duration of 2.5 minutes per bed position.
- **Core Reconstruction Method:** Employs the 3D Ordered Subset Expectation Maximization (3D-OSEM) algorithm modeled with Time-of-Flight (TOF) and Point Spread Function (PSF) (typically 3 iterations and 21 subsets). The image reconstruction matrix is 256 × 256, and a Gaussian post-filter (FWHM 2.0 mm) is applied.

2. 3.0T Liver Multi-parametric MRI Structural and Hemodynamic Acquisition

Acquisition Model: 3.0T superconducting magnetic resonance scanner (Configuration: Siemens Magnetom Vida), equipped with a standard 18-channel body phased-array coil.

2.1 Diffusion-Weighted Imaging (DWI) Sequence

Acquisition Sequence: Free-breathing triggered single-shot spin-echo echo-planar imaging (SS-EPI) sequence.

Core Parameters: TR dynamically adapts to the respiratory rate (typically around 4500 ms), TE is minimized (65 ms), Field of View (FOV) is 380 - 400 mm, matrix is 128 × 128, slice thickness is 3.0 mm, with no interslice gap.

b-value Setting and Mapping: Uses 5 b-values (0, 50, 500, 800, 1000). The scanner workstation automatically generates the apparent diffusion coefficient (ADC) map via voxel-wise fitting using a mono-exponential decay model.

2.2 Contrast-Enhanced MRI (CE-MRI) Temporal Acquisition

Acquisition Sequence: 3D T1-weighted high-resolution volumetric interpolated breath-hold examination sequence (with fat suppression, e.g., Siemens VIBE). TR/TE = 3.4/1.3 ms, flip angle is 9°.

Contrast Agent Administration Strategy: A dual-syringe power injector is used to intravenously bolus standard extracellular fluid contrast agent (Gd-DTPA) via the forearm vein. The dose is 0.1 mmol/kg, and the injection flow rate is strictly set to 2.0 ml/s, followed by a 20 ml saline flush at the same flow rate.

Key Phase Control (Automated Trigger Mechanism):

Pre-contrast Phase (Pre): Breath-hold scan prior to contrast injection.

Arterial Phase (Art): CARE Bolus tracking technology must be used. MR signal threshold, a delay of 5-8 seconds triggers the breath-hold scan (the actual scan center is typically 20-30 seconds post-injection).

Portal Venous Phase (PV): Fixed scan at 60-70 seconds post-contrast injection.

Equilibrium Phase: Fixed scan at 3 minutes post-injection.

Part 2: Data Extraction Workflow and 3D Slicer Preprocessing Pipeline

To transform the underlying hardware data into algorithm-readable matrices, all data must undergo a standardized preprocessing pipeline (an automated workflow built on the 3D Slicer open-source platform).

Spatial Co-registration:

- **Reference Space:** The CE-MRI portal venous phase image is selected as the anatomical reference template (Target).
- **Registration Algorithm:** Utilizes a 6-degree-of-freedom rigid body registration. Based on Mutual Information objective function optimization, the PET (SUV map) and DWI (ADC map) are aligned to the anatomical reference.

Isotropic Resampling:

- Using a 3rd-order B-spline interpolation algorithm, the DICOM matrices of all modalities are uniformly resampled into an isotropic voxel array with absolute physical space consistency (Voxel size: 1.0 mm^3). This step constitutes the basis for ensuring no bias along the X/Y/Z axes for the 3D box-counting algorithm.

Segmentation & Morphological Erosion:

- Senior physicians delineate the 3D region of interest (3D-ROI) slice-by-slice along the tumor margin within 3D Slicer.
- A 3D mathematical morphological erosion operation is applied to the generated 3D-ROI Mask, shrinking it inward by 1 mm (i.e., stripping the outermost voxel). This is to forcefully eliminate edge registration artifacts and partial volume effects caused by respiratory amplitude discrepancies across different modalities.

Part 3: Feature Calculation Principles and Python Core Code Logic

This study relies on underlying Python algorithms to extract FD_PET, FD_ADC, and Afd_MRI. The core computational engine for all features is the 3D Box-Counting Algorithm. **1. Core Mathematical Principle: 3D Box-Counting Algorithm**

- For a given 3D imaging feature matrix, a grid of cubic "boxes" with edge length ϵ is used to cover the target 3D space. The algorithm counts the total number of non-

empty boxes containing the target signal within the ROI mask, denoted as N(ϵ). The Fractal Dimension (FD) is defined as the negative slope of the logarithm of the number of non-empty boxes.

- In actual Python computations, ϵ sequentially takes on widths of 2, 4, 8, 16... voxels. The least squares method is used to fit the linear regression equation of
- $$\mathrm{FD} = \lim_{\epsilon \to 0} \frac{\ln N(\epsilon)}{\ln(1/\epsilon)}$$
- and the slope of this line represents the fractal dimension of the feature. To enhance robustness, the underlying algorithm is set up with loops of random spatial jittering of the grid's initial anchor point, outputting the mean of multiple regression slopes.

2. Metabolic Spatial Fractal Dimension (FD_PET) Extraction Logic

- **Calculation Target:** Spatial distribution heterogeneity of hypermetabolic clones.
- **Data Transformation:** Converts the raw PET radioactivity concentration values into Standardized Uptake Values (SUV).
- $$SUV = \frac{ActivityConcentration \times BodyWeight}{InjectionDose}$$
- **Threshold Filtering:** A mask is applied in Python to extract only the voxel clusters within the tumor ROI with an SUV ≥ 40% of the SUV_max, generating a binarized hypermetabolic 3D point cloud.
- **Fractal Calculation:** This point cloud is input into the 3D Box-counting engine, and the resulting slope is FD_PET.

3. Cytoarchitectural Fractal Dimension (FD_ADC) Extraction Logic

- **Calculation Target:** Spatial complexity of physical matrix collapse (regions of stromal degradation and high cellular density).
- **Discretization Denoising:** To eliminate the baseline drift of absolute ADC values caused by different batches of MRI machines, direct computation on continuous floating-point numbers cannot be performed. The ADC matrix within the ROI must undergo a 64-level equidistant discretization.
- $$ADC_{bins} = digitize(ADC_{raw}, bins = 64)$$
- **Fractal Calculation:** The discretized 3D grayscale topological network is input into the box-counting algorithm, and the output represents the degree of physical barrier disruption, denoted as FD_ADC.

4. Spatiotemporal Perfusion Fractal Acceleration (Afd_MRI) Calculation Logic

- **Calculation Target:** The dynamic spatiotemporal infiltration rate of the microvascular network and the degree of chaos in escape channels.
- **Dimensionality Reduction and Temporal Extraction:** First, structural fractal dimensions are separately calculated for the grayscale images of the three key phases of CE-MRI (pre-contrast phase t_0, arterial phase t_1, portal venous phase t_2), yielding the time series feature vector: FD(t_0), FD(t_1), FD(t_2).
- **Derivative Calculation (Acceleration):** Using time t as the independent variable, the numpy.polyfit function in Python is used to fit the aforementioned discrete points into

a quadratic temporal evolution polynomial.

- $FD(t) = at^2 + bt + c$
- The second derivative with respect to time is calculated for this fitted curve, which is defined as the perfusion fractal acceleration.
- $Afd_{MRI} = \frac{d^2}{dt^2}\mathrm{FD}(t) = 2a$
- (Where the coefficient a is the quadratic coefficient of the polynomial, and the value of 2a signifies the nonlinear "acceleration" of the contrast agent's infiltration rate within the abnormal neovascular network).

Supplementary Table S1. Baseline clinicopathological characteristics of the derivation cohort (n = 100) before and after stabilized inverse probability of treatment weighting (sIPTW).

Variables	Unweighted Derivation Cohort (n = 100)				sIPTW-Weighted Pseudopopulation			
	Low FD_ADC (n = 60)	High FD_ADC (n = 40)	*P* value	SMD	Low FD_ADC	High FD_ADC	*P* value	SMD
Age (years), mean ± SD	62.1 ± 10.5	65.4 ± 9.6	0.112	0.325	63.4 ± 10.2	63.6 ± 10.0	0.865	0.020
Sex, n (%)			0.605	0.103			0.942	0.015
Male	36 (60.0%)	26 (65.0%)			42 (62.5%)	41 (63.0%)		
Female	24 (40.0%)	14 (35.0%)			25 (37.5%)	24 (37.0%)		
Target tumor size, n (%)			0.012	0.518			0.915	0.042
< 3 cm	42 (70.0%)	18 (45.0%)			39 (58.2%)	38 (58.5%)		
≥3 cm	18 (30.0%)	22 (55.0%)			28 (41.8%)	27 (41.5%)		
Number of tumors, n (%)			0.185	0.284			0.880	0.035
Single	32 (53.3%)	15 (37.5%)			31 (46.3%)	30 (46.2%)		
Multiple (≥2)	28 (46.7%)	25 (62.5%)			36 (53.7%)	35 (53.8%)		
Preoperative CEA, n (%)			0.008	0.562			0.924	0.028
< 5 ng/mL	39 (65.0%)	15 (37.5%)			34 (50.7%)	33 (50.8%)		

Variables	Unweighted Derivation Cohort (n = 100)				sIPTW-Weighted Pseudopopulation			
> 5 ng/mL	21 (35.0%)	25 (62.5%)			33 (49.3%)	32 (49.2%)		
Neoadjuvant chemotherapy, n (%)			0.035	0.420			0.955	0.018
No	30 (50.0%)	11 (27.5%)			27 (40.3%)	26 (40.0%)		
Yes	30 (50.0%)	29 (72.5%)			40 (59.7%)	39 (60.0%)		
RAS mutation status, n (%)			0.452	0.155			0.985	0.008
Wild-type	34 (56.7%)	20 (50.0%)			35 (52.2%)	34 (52.3%)		
Mutant	26 (43.3%)	20 (50.0%)			32 (47.8%)	31 (47.7%)		

Abbreviations: FD_ADC, fractal dimension of apparent diffusion coefficient; **sIPTW**, stabilized inverse probability of treatment weighting; **SMD**, standardized mean difference; **CEA**, carcinoembryonic antigen; **SD**, standard deviation.

Note: To strictly prevent data leakage and establish a robust foundation for causal inference, the sIPTW procedure was exclusively performed within the **derivation cohort (n=100).** Continuous variables are presented as mean ± SD and were compared using the Student's *t*-test. Categorical variables are presented as frequencies (percentages) and were compared using the Chi-square test. Before weighting, significant imbalances were observed in target tumor size, preoperative CEA levels, and neoadjuvant chemotherapy history (SMD > 0.10, bolded *P* values < 0.05). After applying sIPTW, the derived pseudopopulation achieved optimal balance across all covariates, strictly bounded within the stringent threshold of SMD < 0.10.

Supplementary Table S2. Comparison of clinical intervention paradigms reflecting the temporal shift between the historical and modern cohorts.

Clinical Intervention Paradigms	Historical Derivation Cohort (2020–2023, n = 100)	Modern Validation Cohort (2024–2025, n = 45)	*P* value
Neoadjuvant chemotherapy, n (%)			0.012
No	58 (58.0%)	16 (35.6%)	
Yes	42 (42.0%)	29 (64.4%)	
Targeted therapy (e.g., Bevacizumab/Cetuximab), n (%)			0.007
No	74 (74.0%)	23 (51.1%)	
Yes	26 (26.0%)	22 (48.9%)	
Preoperative Objective Response Rate (ORR), n (%)*			0.018
Non-responders (SD/PD)	65 (65.0%)	20 (44.4%)	
Responders (CR/PR)	35 (35.0%)	25 (55.6%)	

Abbreviations: ORR, objective response rate; **SD**, stable disease; **PD**, progressive disease; **CR**, complete response; **PR**, partial response.

Note: Categorical variables are presented as frequencies (percentages) and were compared using the Chi-square test. *The preoperative ORR was evaluated according to RECIST 1.1 criteria. This table quantitatively demonstrates a significant "temporal shift" in real-world clinical practice: patients in the modern validation cohort (2024-2025) received a significantly higher intensity of systemic treatments (neoadjuvant chemotherapy and targeted therapy) and achieved a higher ORR compared to the historical derivation cohort (2020-2023). Despite

these profound shifts in background treatment paradigms and macroscopic tumor shrinkage, the FRAC-3D model maintained highly robust predictive performance, verifying its capacity to capture the fundamental biophysical drivers of MVI independent of external chemical stressors.

Supplementary Figures

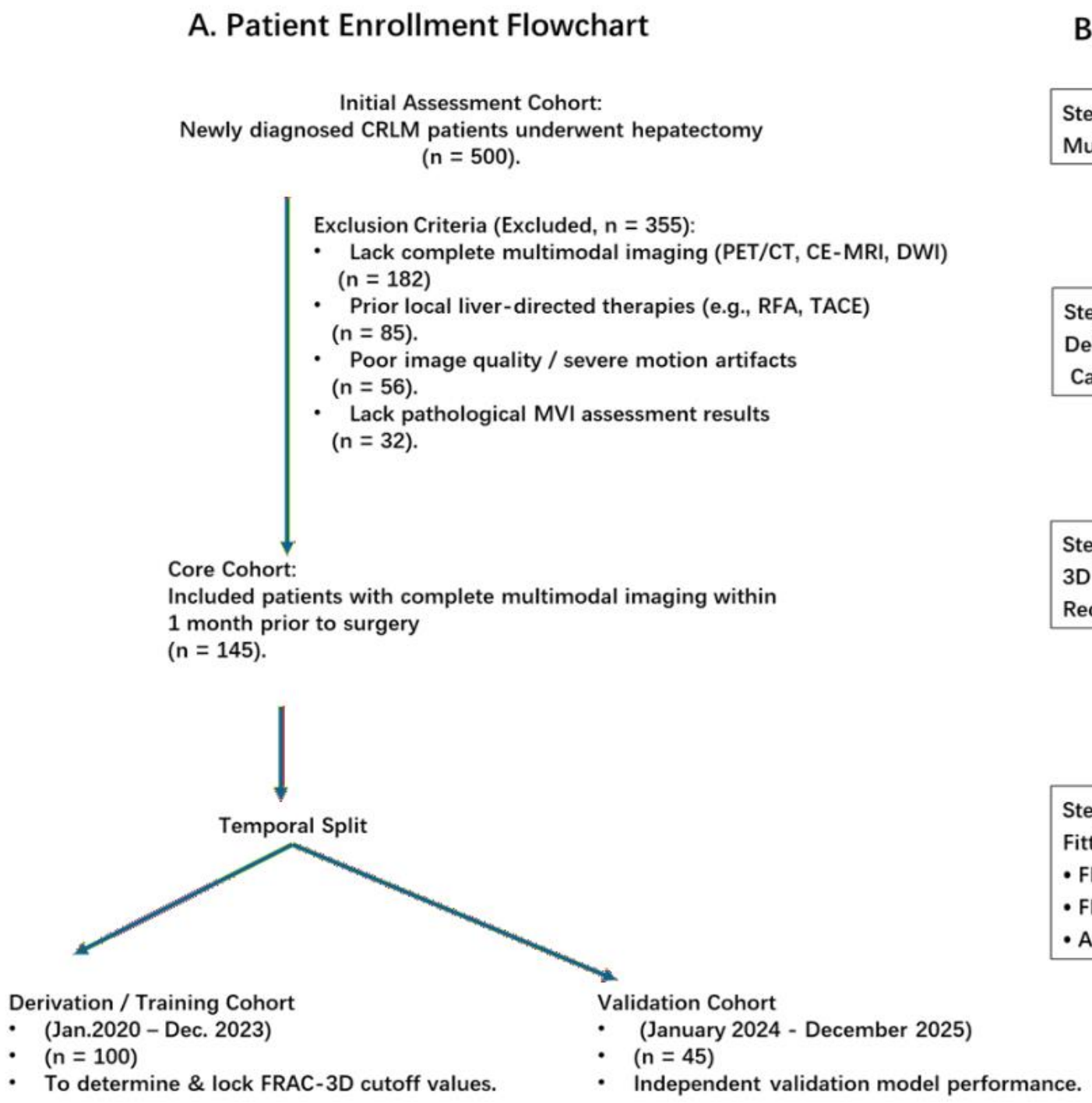

Supplementary Figure S1. Patient selection flowchart and the FRAC-3D image processing pipeline.

(A) Flow diagram of patient enrollment and exclusion criteria. A total of 500 patients with CRLM were initially screened. After applying rigorous exclusion criteria, a core cohort of 145 patients with complete multimodal imaging (PET/CT, CE-MRI, and DWI) and pathological MVI confirmation was established. This cohort was temporally divided into a training set (n = 100, for threshold locking) and an independent validation set (n = 45). **(B)** The standardized FRAC-3D feature extraction pipeline. Raw DICOM images underwent spatial registration and isotropic resampling (1.0 mm³). Following 3D volume of interest (VOI) segmentation, the spatial heterogeneity of the tumor microenvironment was quantified using a 3D box-counting algorithm. The multidimensional fractal features (FD_PET, FD_ADC, and Afd_MRI) were mathematically derived by calculating the negative slope of the log-log plot of the box size (ϵ) versus the number of non-empty boxes N(ϵ).

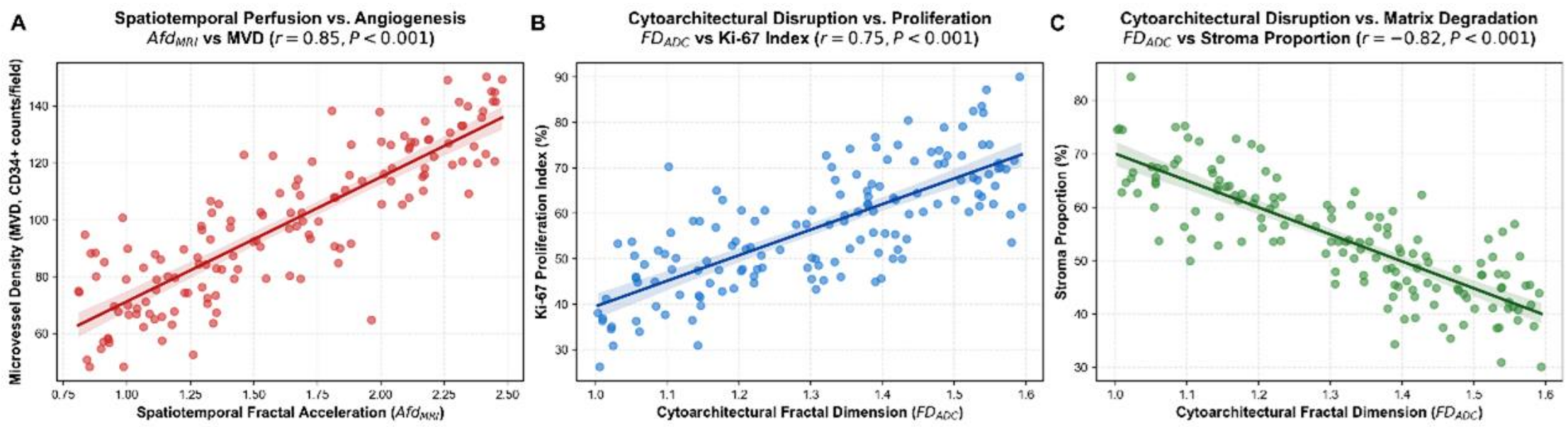

Supplementary Figure S2. Cross-scale radiologic-pathologic mapping of patient-level biophysical surrogate correlations for the FRAC-3D signature.

(A-B) Representative cases of Phenotype I (Static-Ordered, low FD_ADC) and Phenotype III (Highly Chaotic, high FD_ADC). Corresponding histologic sections (H&E) and immunohistochemical (IHC) staining for CD34 and Ki-67 are displayed. Compared to Phenotype I, Phenotype III tumors exhibit significantly higher microvessel density (MVD), marked stromal depletion, and extreme cellular proliferation levels. **(C)** Scatter plots with linear regression lines demonstrating the correlation between the architectural fractal dimension (FD_ADC) and pathological markers across the entire cohort (n = 145). Strong positive correlations were observed between FD_ADC and both the Ki-67 proliferation index (r = 0.748, P < 0.001) and CD34-positive MVD (r = 0.675, P < 0.001). These results validate that the macroscopic spatiotemporal fractal dynamics derived from FRAC-3D can serve as reliable biophysical surrogates reflecting microscopic tumor aggressiveness and microenvironmental remodeling.
