# Supplementary material for: Three-dimensional multimodal fractal dynamics integrating spatial metabolism, cytoarchitecture, and perfusion for preoperative prediction of microvascular invasion in colorectal liver metastases
Source: J Transl Med. 2026 Jul 27;24:1335. doi: 10.1186/s12967-026-08712-y (PMC13692898; doi:10.1186/s12967-026-08712-y)
Supplement: Supplementary file 2 [file 12967_2026_8712_MOESM2_ESM.docx]

TRIPOD Statement: Checklist for Prediction Model Development and Validation

| **Section/**  **Topic** | **Item** | **Checklist Item** | **Content / Location in "manuscript_final.docx"** | **Page** |
| --- | --- | --- | --- | --- |
| **TITLE & ABSTRACT** |  |  |  |  |
| Title | 1 | Identify the study as developing and/or validating a multivariable prediction model, target population, and outcome. | Title: "Three-Dimensional Multimodal Fractal Dynamics (FRAC-3D)... for Preoperative Prediction of Microvascular Invasion in Colorectal Liver Metastases". | 1 |
| Abstract | 2 | Summary of objectives, design, setting, participants, predictors, outcome, analysis, results, and conclusions. | Abstract section: Includes objectives (n=145/45), AUC 0.938, and MVI prediction conclusions. | 1 |
| **INTRODUCTION** |  |  |  |  |
| Background | 3a | Explain medical context and rationale for the model, including references to existing models. | Introduction: Discusses MVI importance, Euclidean geometry limitations, and preliminary FRAC-V dual-modal model. | 3 |
| Objectives | 3b | Specify objectives, including whether development, validation, or both. | Introduction (last paragraph): Integration of PET/CT, MRI, and DWI for development and temporal validation of FRAC-3D. | 5 |
| **METHODS** |  |  |  |  |
| Source of data | 4a | Describe study design or source of data separately for development and validation sets. | Methods: Single-center, retrospective nested cohort study using a prospectively maintained database. | 5 |
|  | 4b | Specify key study dates (accrual and follow-up). | Materials and Methods: Accrual between January 1, 2020, and December 31, 2025. | 6 |
| Participants | 5a | Specify study setting and number of centers. | First Affiliated Hospital of Jinzhou Medical University (Single center). | 5 |
|  | 5b | Describe eligibility criteria for participants. | Study design and participants: 4 inclusion and 4 exclusion criteria listed. | 6 |
|  | 5c | Details of treatments received. | Table 1 and Methods: R0 hepatectomy, neoadjuvant chemotherapy, and targeted therapy. | 6 |
| Outcome | 6a | Define the outcome, how and when assessed. | Outcomes: Histopathologically confirmed MVI via H&E and CD34 staining. | 9 |
|  | 6b | Blind assessment of the outcome. | Outcomes: Pathological slides independently adjudicated by two pathologists double-blinded to model results. | 9 |
| Predictors | 7a | Define all predictors, how and when they were measured. | Spatial fractal extraction: FD_PET (metabolism), FD_ADC (architecture), and Afd_MRI (perfusion). | 8 |
|  | 7b | Blind assessment of predictors. | Procedures: ROIs delineated by radiologists blinded to clinical and pathological data. | 7 |
| Sample size | 8 | Explain how study size was arrived at. | Study design: 500 patients screened; core cohort of 145 patients with complete tri-modal imaging. | 6 |
| Missing data | 9 | Describe how missing data were handled (e.g., multiple imputation). | Statistical analysis: Multiple Imputation by Chained Equations (MICE) used for minor missing data (<5%). | 9 |
| Statistical analysis | 10a | Describe how predictors were handled. | Statistical analysis: Multi-factor regression; thresholds (e.g., 1.35 for FD_ADC) locked in derivation cohort. | 10 |
|  | 10b | Specify model type, building procedures, and internal validation. | Logistic regression for development; temporal validation for model testing. | 10 |
|  | 10d | Measures used to assess/compare model performance. | AUC, calibration curves, Hosmer-Lemeshow test, continuous NRI, IDI, and DCA. | 10 |
| Risk groups | 11 | Details on how risk groups were created. | Survival analysis: Unsupervised K-Means clustering into Phenotypes I, II, and III. | 11 |
| **RESULTS** |  |  |  |  |
| Participants | 13a | Flow of participants and number with/without outcome. | Results 3.1: 145 total core patients; 55 (37.9%) confirmed MVI-positive. | 11 |
|  | 13b | Characteristics of participants and available predictors. | Table 1: Baseline comparison of derivation (n=100) and validation (n=45) cohorts. | 12 |
| Model development | 14a | Number of participants and outcome events in each analysis. | Derivation cohort (n=100, 38 MVI+) and Validation cohort (n=45, 17 MVI+). | 12 |
|  | 14b | Unadjusted association between candidate predictors and outcome. | Table 2: Univariable Analysis results provided for clinical and fractal variables. | 45 |
| Model performance | 16 | Performance measures (with CIs) for the model. | Results 3.3: Validation AUC 0.938 (95% CI: 0.89–0.97); NPV 96.2%. | 13 |
| **DISCUSSION** |  |  |  |  |
| Limitations | 18 | Discuss any limitations of the study. | Discussion 4.5: Sample size, single-center design, cross-device heterogeneity, and registration errors. | 24 |
| Interpretation | 19b | Overall interpretation considering objectives and limitations. | Discussion 4.1: Cross-validation of tri-modal features; synergetic "metabolism-physics-hemodynamics" coupling. | 18 |
| Implications | 20 | Potential clinical use and future research. | Discussion 4.3: Precision surgical planning (PSH vs. Extended margins) and neoadjuvant strategy. | 22 |
| **OTHER** |  |  |  |  |
| Supplementary info | 21 | Availability of protocol, datasets, and resources. | Data and Code Availability: Datasets and Python scripts available from corresponding author upon request. | 33 |
| Funding | 22 | Source of funding and role of funders. | Funding: Supported by GDUPT Talent Recruitment Projects (Nos. 2024rcyj1055, 2024rcyj1056). | 32 |
